# Can Social Norms Promote Recycled Water Use on Campus? The Evidence From Event-Related Potentials

**DOI:** 10.3389/fpsyg.2022.818292

**Published:** 2022-02-04

**Authors:** Xiaojun Liu, Shiqi Chen, Xiaotong Guo, Hanliang Fu

**Affiliations:** ^1^School of Management, Xi’an University of Architecture and Technology, Xi’an, China; ^2^Key Research Bases for the Co-construction and Sharing for Human Settlement Environment and Good Life of the New Era in Shaanxi, Xi’an, China; ^3^Laboratory of Neuromanagement in Engineering, Xi’an University of Architecture and Technology, Xi’an, China

**Keywords:** social norms, recycled water, public willingness, feedback-related negativity, social distance

## Abstract

The unwillingness of college students to use recycled water has become a key barrier to sewage recycling on campus, and it is critical to strengthen their inclination to do so. This paper used college students in Xi’an as a case study and adopted event-related potential technology to explore the effect of social norms on the willingness to use recycled water and the neural mechanism of cognitive processing. The results suggested the following: (1) The existence of social norms might influence college students’ willingness to use recycled water. (2) When individuals’ willingness to use recycled water is lower than the social norm, there is a bigger feedback-related negative amplitude. (3) College students pay more attention to social norms in groups with closer social distance. These findings can be used to provide a scientific basis for persuading the public to use recycled water from the perspective of the social norm to drive public acceptability.

## Introduction

By 2040, two billion people will be affected by the global groundwater crisis (Catley-Carlson, 2019; Tortajada and van Rensburg, 2020), as a result, forcing more and more cities to seek unconventional water sources. Sewage recycling is one of the most commonly used unconventional water sources. It alleviates water environmental pollution, the current challenge of water scarcity, and increases water resource use efficiency by re-developing and utilizing urban domestic sewage treatment after reaching the standard.

Many countries have been exploring recycled water treatment technology for a long time, and the utilization rate of recycled water has been included in the green building index and urban development planning index (Lee and Shepley, 2019; Lwin and Panuwatwanich, 2021). Currently, the recycled water treatment technology can almost produce recycled water that meets any water quality standards, and the technical issues are no longer an obstacle to its utilization (Fielding et al., 2019). The public’s mistrust, prejudice, and rejection of recycled water have become the major roadblocks to the promotion of recycled water. Given the importance of public participation in the implementation of new technologies or policies (Guo et al., 2021), enhancing the public’s willingness to use recycled water has become a pressing issue to be addressed.

Incentive theory proposes that the target population can be intervened externally through material and spiritual incentives to influence their behavior toward the intended outcome. In sewage regeneration and usage, using recycled water can reduce water costs by lowering the consumption of tap water, which is considered a material incentive (Deh-Haghi et al., 2020). When the price difference between recycled water and tap water is larger, the motivating power for potential recycled water users is stronger (Garcia-Cuerva et al., 2016).

However, in China, this material incentive has not been enough to persuade the public to use recycled water, mainly for two reasons: on one hand, China has had a long time implementation of the low-price policy for tap water, which often fails to reflect the scarcity of water (Mu et al., 2019). Even in some areas, meeting the development and operation expenditures of water conservancy facilities remains a challenge. On the other hand, the high costs of production and operation facilities for recycled water, as well as the cost input into the production process, limit the amount of room for the recycled water price reduction. Therefore, in China, it is difficult to implement a driving strategy to encourage the public to use recycled water through price differences.

Hence, more research has changed the focus to explore how spiritual motivation might be used to drive an individual to use recycled water (Liu et al., 2018; Xue et al., 2020). Social norms, which depend on individuals social characteristics and have an incentive influence on individual behavior through the external social environment of individuals, are considered to be one of the important variables affecting the public acceptability of recycled water use (Li et al., 2021). Existing studies mostly use social norms as auxiliary factors to explore the effect of social capital (Dean et al., 2016), conformity (Leong and Lebel, 2020), attitudes toward water sources (Mankad et al., 2019), and other influencing factors on the public’s willingness to accept recycled water. However, from a cognitive level, there is no clear conclusion about whether social norms can promote public usage of recycled water. This study examines the neurological effects of social norms on campus recycled water use to further analyze the role of social norms in promoting recycled water projects.

## Theoretical Background and Research Questions

### The Guiding Effect of Social Norms on Individual Behavior

The concept of social norms originated in the sociological research field and has since been widely applied in neuroscience, management, psychology, and a variety of other fields, it conveys which opinions or behaviors can be accepted or agreed upon by a certain group. Social norms are different from moral norms, legal norms, personal norms, and habitual behaviors, which provide guidance or constraints for individual behaviors (Elster, 1989). Studies have shown that social norms have a significant impact on promoting pro-environmental behavior such as the use of recycled water (Hou et al., 2020). During the interaction between individuals and society, individuals will be strongly influenced by the attitudes and views of members around them on recycled water usage. Therefore, social norms have an important role in individuals’ willingness to reuse recycled water.

### Social Distance Leads to Heterogeneous Social Norms

The guiding effect of social norms on individual behavior or willingness has been demonstrated in many aspects, but the heterogeneity of the influence effect of social norms is often neglected. This heterogeneity is often caused by the social distance of the groups that establish social norms. The concept of social distance is beneficial for determining the level of intimacy between individuals and enhancing the impact of social emotions (Joo et al., 2018). Individual behaviors are often guided by social norms produced by different social distance groups (Lo et al., 2019). For pro-environmental behavior, the social distance attribute of the group that develops social norms will directly or indirectly affect the final acceptance effect of individuals to social norms (Huang et al., 2021). The difference in the impact of social distance on individuals is reflected in the transformation from self-identity to group identity. The social norms proposed by the social groups recognized by individuals could encourage everyone to strive toward a common goal (Kim et al., 2017). Hence, the heterogeneity of individuals under the influence of social norms created by different social distance groups must also be considered.

### Event-Related Potentials Relate to Social Norms and Social Distance

Recently, scholars have gradually begun to pay attention to how individuals process acquired social norms cognitively at the neural level, and the guiding effect of social norms on individual behavior after cognitive processing (Du et al., 2019; Wang et al., 2019). Compared to other technologies, event-related potentials (ERPs) have been widely used in the study in cognitive neuroscience, due to its advantages of high time resolution, low cost, and ease of access (Hou et al., 2021), it makes a certain contribution for exploring individual’s insight, emotion, and cognition of social norms. Therefore, ERPs experimental methodologies will also be applied in the study.

#### Feedback-Related Negativity Component

The research found that the anterior insula (AI) and anterior cingulate cortex (ACC), which sense motivational conflict and process negative emotions, were activated by norm violations (Rawls and Lamm, 2021). Feedback-related negativity (FRN), a component of ERPs derived from AI and ACC sites, was found to measure individual evaluation of outcomes (Holroyd and Coles, 2002). The FRN component is a steeply alternating positive-negative-positive waveform that typically peaks between 200 and 350 ms after stimulation (Sambrook and Goslin, 2015). It is often observed when there is a sense of result conflict. The FRN component is regarded as a reliable event-related potential component in social neuroscience, particularly in terms of monetary loss and moral perception, and it is sensitive to negative feedback such as damage to interests and violation of social norms (Lu et al., 2020). Higher levels of FRN activation are often accompanied by a proclivity to pay close attention to existing social norms. Therefore, the FRN component would be selected as one of the representations in this study to portray college students’ perceptual conflicts due to violation of social norms.

#### P300 Component

After the induction of FRN, several studies have also discovered that behaviors that cause a sense of violation of rules or expectations can induce the related subsequent P300 component (Niedeggen et al., 2019; Porcaro et al., 2019; Zhan et al., 2020). The P300 component that appears after the FRN amplitude peak usually reaches its maximum positive amplitude 200–600 ms after stimulation. The presence of this component is usually linked to the allocation of attentional resources among participants. Existing studies have suggested that the larger the amplitude of P300, the more attention is allocated to a particular task (Du et al., 2019). At the same time, in the “Judge-Advisor System” scenario commonly used to explore the influence of social norms, P300 has been confirmed to be related to the behavioral adjustments of individuals when they are aware of group opinions (Wang et al., 2019). Therefore, P300 will be used in this study as another representation of perceived conflict caused by violations of social norms among college students.

### Research Questions

This study aims to help people better understand how social norms promote the use of recycled water on campus, using college students in Xi’an as a case study. We provided several suggestions to promote the use of recycled water on campus according to the research results. Specific research questions include:

(1) What kind of expression of social norms can better elicit the resonance of individuals?
(2) When individuals diverge from social norms, what attitudes will they have?
(3) What attitudes will individuals have when confronted with social norms proposed by different social distance groups?

## Materials and Methods

### Experimental Tasks

The experimental tasks in this study included two stages: social norms scenario activation experiment and event-related potentials experiment.

The social norm scenario activation experiment was mainly carried out in the form of a questionnaire. The respondents were required to read social norm activation material. After confirming the social norm activation effect, the six-point Likert scale was used to collect the respondents’ acceptance intention for 12 types of recycled water. According to the reading material, the respondents were divided into “no social norms group,” “descriptive social norms activation group,” and “injunctive social norms activation group.” After reading the activation materials, respondents need to set corresponding questions to test the effect of social norm activation. Then the questionnaire data failing to activate social norms should be eliminated. According to the investigation results of this stage, a more effective presentation of social norms in ERP experiment was selected. Bringing experimental participants into the context of ERP experiment is an important purpose of taking social norm scenario activation experiment as pre-experiment. The false report method adopted in the research on college students’ energy-saving behaviors by Hamon and Smith (2016) will be used to present social norms in the ERP experiment. In fact, these presented social norms are artificially manipulated by the experimental design. Therefore, in order to improve the credibility and effectiveness of stimulus materials presented in the ERPs experiment, it is necessary for participants to feel their willingness to participate in recycled water and those around them in advance.

**FIGURE 1:**
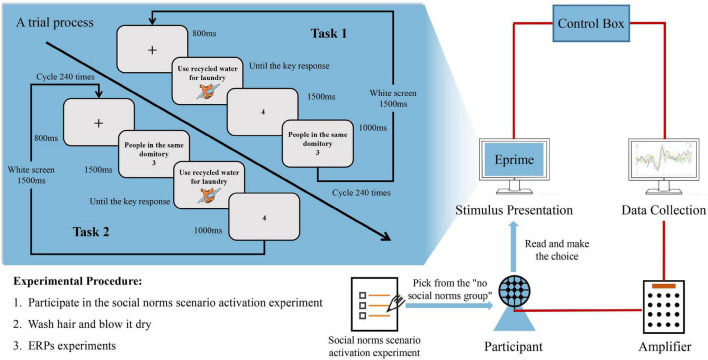
Experiment procedure (The text-type stimulus materials were presented in Chinese in the formal experiment).

The second stage was to conduct an ERP experiment on some of the “no social norms group” participants. The ERP experiment task of this study was adapted from the classic Judge-Advisor System (JAS) paradigm (Sniezek and Buckley, 1995). College students in this study would function as policymakers and choose the willingness to accept recycled water reuse as a decision task. The false report method was used to present the social norms created by different groups as suggestions for the subjects’ reference. In addition, in this experiment, the difference between the decision maker’s initial willingness value of recycled water usage and the presented social norm value was defined as the social norm deviation value. There is no social norm deviation when the value of social norm deviation is zero, in the opposite, there is social norm deviation. When social norm deviates, it is separated into two categories: positive social norm deviation and negative social norm deviation. Positive social norm deviation occurs when the social norm value presented exceeds the decision maker’s initial willingness value of recycled water usage. Conversely, it is defined as negative social norm deviation. Different from the initial decision-making, others’ suggestions, and final decision-making contained in the traditional JAS paradigm process, this study divided the advice-taking process into two tasks. Both tasks were repeated 240 times. In Task 1, the participants were informed about the social norms of the people around them after the participants made the initial choice of reclaiming water. In Task 2, the order of the trial will be reversed. Participants will first be informed about the social norms of people around them before being asked about their willingness to use recycled water. Prior to the experiment, the participants would be notified by experimental instructions that they would be taking part in a study on the willingness to use recycled water. Participants will then be instructed to press the space bar to proceed with the actual experiment after confirming the understanding of the experimental task. Before each trial of Task 1 starts, a “ + ” would be displayed in the center of the screen for 800 ms to focus the participants’ mental energy. On the next page, the screen is randomly selected from six directions of recycled water reuse, participants would make their willingness choice on this interface. After presenting the results of participants’ choice of willingness to reuse recycled water in 1,000 ms, the social norms of a certain social group in 1,500 ms would be presented. The social norms presented in this interface are manipulated so that the difference between the values of the social norms presented and the values of the participants’ willingness are as follows: 0 (80 trials), + 1 (26 trials), −1 (26 trials), + 2 (27 trials), −2 (27 trials), + 3 (27 trials), and −3 (27 trials) were evenly divided into three social distance groups. After a trial, the screen would be blank for 1,500 ms. The trial would be carried out 240 times. After Task 1 was completed, there would be a 5-min break before Task 2 began. The difference between the trial in Task 1 and Task 2 is the presentation of social norms placed before the participants’ willingness choices. At the same time, the presented social norms were manipulated and randomly presented into 1 (40 trials), 2 (40 trials), 3 (40 trials), 4 (40 trials), 5 (40 trials), and 6 (40 trials), with each trial evenly divided into three social distance groups. This trial would also be carried out 240 times. The schematic information of the experimental structures of each trial in Task1 and Task 2 are shown in Figure 1. More detailed event-related potential experiment procedures for this study are presented in the Supplementary Material, and the original meaning of all abbreviations in the article can be found in Appendix A.

### Participants

The participants in the social norm scenario activation experiment were college students from Xi’an University of Architecture and Technology. Questionnaires were distributed by random interviews. If the respondents were willing to participate in the ERPs Experiment, they would be given priority to fill in the questionnaire of the “no social norm group.”

Then, thirty college students from Xi’an University of Architecture and Technology (15 males, 15 females; mean age: 23.14 ± 1.77 years) were recruited as participants in the formal event-related potential experiment. All participants were right-handed, had normal vision or corrected vision, had no history of neurological disease or genetic history, and had not previously participated in a similar experiment. All participants have filled in written informed consent before the experiment, and the study will strictly abide by the Declaration of Helsinki (General Assembly of the World Medical Association, 2014). The Internal Review Board of the Laboratory of Neuromanagement in Engineering approved this study.

### Stimulative Materials

The independent sample *T*-test was used to determine the difference of the effects of descriptive social norms and injunctive social norms on college students’ willingness to reuse recycled water. The results show that college students’ willingness to reuse recycled water under the influence of descriptive social norms (4.631 ± 0.731) is higher than that under the influence of injunctive norms (4.234 ± 0.617), the independent sample *T*-test results showed that there was a statistical difference between the two (*T* = 2.016, *P* = 0.003). Descriptive social norms have a better activation effect on college students in the scenario of recycled water reuse. Therefore, descriptive social norms are selected as the presentation method of stimulus materials in ERPs experiment.

In the ERP experiment, the mismatch between the expected feedback and the actual feedback is one of the preconditions to induce FRN components (Li et al., 2018). In order to create a social norm deviation scenario, the first six reuse directions with large variance values were selected as stimulus materials for the ERP experimental task from the twelve types of recycled water reuse directions provided by the situational activation experiment (Variance goes from large to small: water the vegetables, take a shower, clean hands, wash clothes, brush teeth, drink).

This experiment assumes that the social distance of college students in the social group from far to near is the same school, the same major, and the same dormitory. At the same time, in order to ensure the credibility of social norm deviation scenarios, extreme value deviation should be avoided when presenting social norms. In this study, the range of the value deviation of descriptive social norms presented by participants after their initial willingness selection in Task 1 was controlled within ± 3. The stimulus materials involved in the event-related potential experiment of this study can be found in Supplementary Material.

### Electroencephalography Data Collection and Analysis

This experiment was conducted in the Laboratory of Neuromanagement in Engineering, Xi’an University of Architecture and Technology. Experimental programming and presentation were implemented *via* E-Prime3.0. The experiments were divided into two phases: Task 1 (240 Trials) and Task 2 (240 Trials). Normally, the duration of each task is between 25 and 30 min. In order to ensure the participants’ concentration during the experiment, a mandatory rest time of 5 min was set between two tasks. Before the ERP experiment, participants should come to the lab at least 45 min in advance to cooperate with the researchers to prepare for the experiment and inject electro-gel into each electrode on the electrode caps to reduce its resistance to less than 5 kΩ. In the formal experiment, participants were asked to watch the 22-in Dell Monitor, Dell Technologies, Texas, TX, United States, with a refresh rate of 60 Hz and a resolution of 1,920 × 1,080 pixels in soft indoor light at a visual distance of 100 cm. Experimental EEG signals were recorded using a Neuroscan Synamps2, Compumedics Neuroscan, North Carolina, NC, United States, amplifier connected to an elastic cap with 64 Sintered Ag/AgCl electrodes. The sampling rate was 1,000 Hz, GND and REF were the reference grounding electrodes, and the signals of bilateral mastoid electrodes were referenced. EEG signals are transmitted with a 0.01–70 Hz bandpass. The horizontal electrooculogram was recorded by placing the HEO electrode 1 cm behind the tail of both eyes while the vertical electrooculogram was recorded by placing the VEO electrode 1 cm above and below the left eye. In order to reduce the interference of blinking and other eye movements to electroencephalography signals, the aboveelectrooculogram data would be algorithmically corrected in Curry 8 processing. Artifact removal was performed based on baseline correction and EOG removal, electrical signals with amplitude greater than 100 μV would be regarded as bad blocks and will be ignored in the subsequent analysis. This experiment only focused on the EEG signal changes of participants after the introduction of social norms, therefore, the EEG fragments from 200 ms before the introduction of social norms to 700 ms after the introduction of social norms were intercepted. Combined with the final waveform of the experiment and previous studies, FRN and P300 components were chosen to indicate the influence of social norm deviation caused by different social distance groups. The amplitude of FRN was measured at FZ, FCZ, and CZ electrodes (Zioga et al., 2019), and the amplitude of P300 was measured at CZ, CPZ, and PZ electrodes (Yu et al., 2019).

In this study, in order to confirm college students’ perceptions of social norm deviation scenarios, repeated-measures ANOVAs were used to verify the peak amplitude data of FRN and P300 in Task 1. In order to confirm the impact of social norm deviation scenarios produced by different social distance groups on the willingness of college students to reuse recycled water [2 (Social norm deviation: positive social norm deviation and negative social deviation) × 3 (Social distance: people in the same dormitory, people in the same major and people in the same school)], two-way ANOVA was used to verify the peak amplitude data of FRN and P300 in Task 1. Finally, the P300 amplitude peak data in Task 2 was verified by repeated-measures ANOVAs to supplement the cognitive attention of social distance among college students.

## Results

### Perception of Social Norm Deviation

**FIGURE 2:**
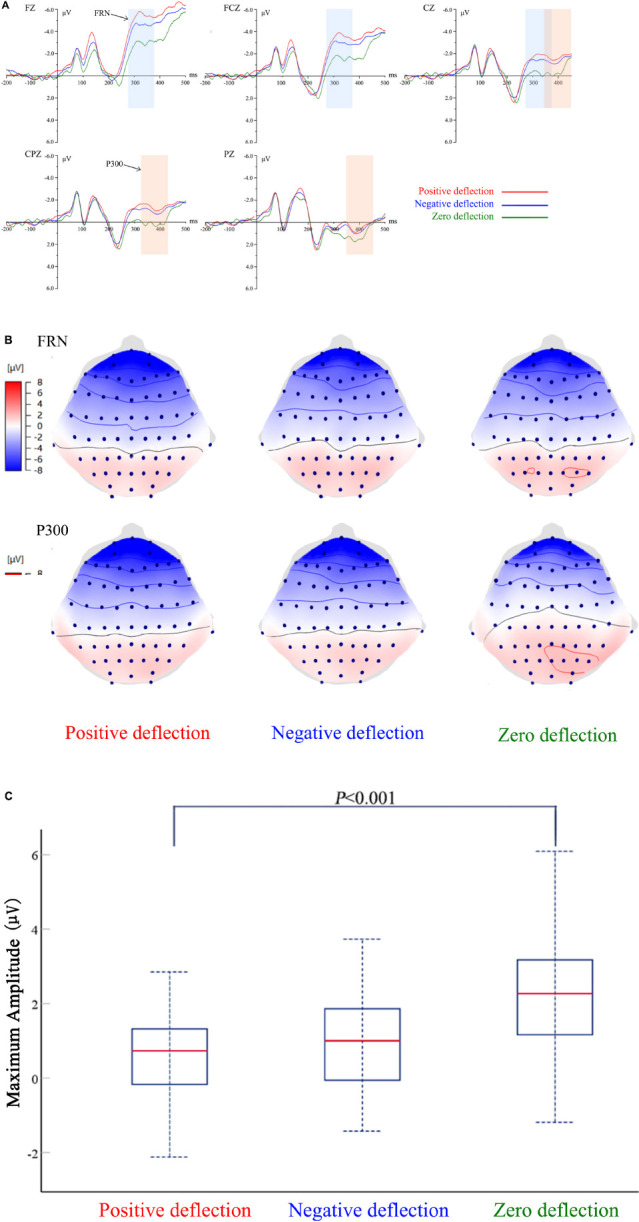
**(A)** The grand averaged event-related potentials (ERPs) waveforms at FZ, FCZ, CZ, CPZ, and PZ of social norm deviation in Task 1. **(B)** The scalp distribution of feedback-related negativity (FRN) and P300 of social norm deviation in Task 1. **(C)** ANOVA results of social norm deviation in Task 1.

In order to explore college students’ perception of social norm deviation in recycled water usage, the difference between participants’ social norm situation and their willingness to use recycled water was divided into three categories: no social norm deviation, positive social norm deviation, and negative social deviation. Figure 2A shows the average ERPs waveform induced on FZ, FCZ, CZ, CPZ, and PZ electrodes in the above three scenarios. Figures 2B,C show the topographic maps and peak variance analysis of FRN and P300. Table 1 shows the average peak value and standard error of FRN and P300 in the above three scenarios.

**TABLE 1:** The average peak value and standard error of FRN and P300 in the social norm deviation scenario.

Social norm deflection scenario	EEG components			
	FRN		P300	
	Average peak value	Standard error	Average peak value	Standard error
Positive deflection	−4.227	2.184	0.613	1.150
Negative deflection	−3.392	2.842	0.990	1.310
Zero deflection	−1.985	2.798	2.330	1.650

#### Feedback-Related Negativity

The stronger the participants’ perceived motivation and emotion for the feedback results, the greater the amplitude of FRN induced. In this study, the EEG amplitude observed on FZ, FCZ, and CZ electrodes between 280 and 380 ms were observed as the FRN component. The amplitude reflected the views and attitudes of college students when their willingness was inconsistent with social norms. By comparing the FRN in the waveform, it can be observed that if there is a deviation between the willingness of self-recycled water reuse and the social norm, the FRN amplitude would most likely be more significant than without social deviation (*M* = −1.985, SE = 2.798). A repeated-measures ANOVAs result (*F* = 5.588, *P* = 0.005) reveals the above results. In other words, participants perceived their level of willingness to use recycled water as incorrect when the opinions around them differed from their own. In the deviation scenario, the positive deflection of the social norm (*M* = −4.227, SE = 2.184) induced a stronger FRN amplitude than the negative deflection of the social norm (*M* = −3.392, SE = 2.842). It was shown that positive social norm deviation in the use of recycled water could lead to a more erroneous perception among college students.

#### P300

The P300 component was related to the allocation of attention resources, and the larger the amplitude of the P300 component was, the more attention participants allocated. The P300 is also more sensitive to positive feedback. In this study, the P300 component was determined by measuring the EEG amplitude observed at CZ, CPZ, and PZ electrodes between 350 and 450 ms. The amplitude represented the degree of individual attention to different situations when college students’ willingness and social norms were different. A repeated-measures of ANOVAs result of P300 amplitude revealed the primary effect of social norm deviation. When one’s own choice was consistent with the choice of the surrounding people (*M* = 2.330, SE = 1.650), P300 would be more significant than the situation of social norm deviation. However, the independent sample *T*-test results revealed that there was no significant difference in P300 amplitude between positive social norm deviation and negative social norm deviation (*F* = 0.940, *P* = 0.336).

### Perception of Social Norm Deviation Generated by Different Social Distance Groups

In order to further explore college students’ attitudes toward social norms proposed by different social distance groups, we analyzed the FRN component (280–380 ms) and P300 component (350–450 ms), respectively, according to the FZ, FCZ, and CZ electrode waveforms in Task 1 and CZ, CPZ, and PZ electrode waveforms in Task 2. Table 2 shows the average peak values and standard errors of FRN and P300 in the following three types of social distance: same dormitory, same major, and same school.

**TABLE 2:** The average peak value and standard error of FRN and P300 in different social distance scenarios.

Social norm scenario		EEG components			
Social norm deflection	Social distance	FRN		P300	
		Average peak value	Standard error	Average peak value	Standard error
Dormitory	Positive deflection	−6.616	2.104	1.920	1.343
	Negative deflection	−5.193	1.911		
Major	Positive deflection	−4.849	2.219	1.302	1.669
	Negative deflection	−4.380	2.251		
School	Positive deflection	−3.934	2.538	0.959	1.090
	Negative deflection	−3.523	2.415		

#### Feedback-Related Negativity

**FIGURE 3:**
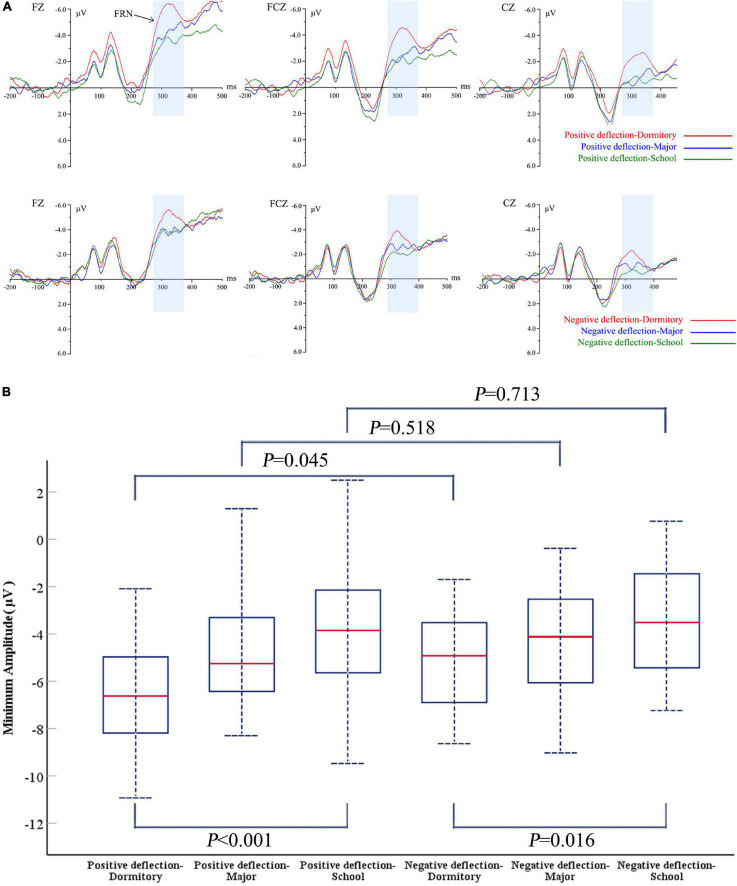
**(A)** Grand averaged ERPs waveforms at FZ, FCZ, and CZ of social norm deviations from different social distances in Task 1. **(B)** ANOVA results of social norm deviations from different social distances in Task 1.

Figure 3A shows the average ERPs waveform of FRN induced by Task 1 at FZ, FCZ, and CZ electrodes for three social distance groups under positive social norm deviation and negative social norm deviation, respectively.

In Figure 3B, the FRN amplitude awakened by different social distances is always statistically significant in both positive and negative social norm deflection scenarios (positive deviation: *F* = 10.507, *P* < 0.001, negative deviation: *F* = 4.315, *P* = 0.016). In other words, when others’ social norms are inconsistent with their own choices, college students pay different attention to the social norms proposed by different social distance groups. Figure 3A shows that compared with the people of the same major and the same school, the willingness of recycled water reuse of dormitories has the most obvious effect on the excitation of FRN amplitude (positive deviation: *M* = −6.616, SE = 2.104, negative deflection: *M* = −5.193, SE = 1.911). However, the value of FRN amplitude of social norms produced for people in the same major (positive deviation: *M* = −4.849, SE = 2.219, negative deflection: *M* = −4.380, SE = 2.251) is slightly lower than that of people in the same school (positive deviation: *M* = −3.934, SE = 2.538, negative deflection: *M* = −3.523, SE = 2.415). The results support that social distance will affect the perceptual sensitivity of individual choice when it is inconsistent with group choice.

In Figure 3B, the social distance was used as a variable to compare the cognition of positive and negative social norm deviations of college students in the same group. After the ANOVA test, only the FRN amplitude of social norms in the same dormitory under different deviation scenarios was statistically significant (*F* = 4.194, *P* = 0.045). As shown in Figure 3A, positive social norm deflection causes larger FRN waves than negative social norm deflection. In other words, when dormitory mates have more positive intentions for using recycled water than their own, college students will have a more obvious false perception of their own choice. While when others have fewer intentions of using recycled water, their false perception will be significantly weakened. However, in the analysis of variance, the above results could not be confirmed in the same major group (*F* = 0.423, *P* = 0.518) and the same school group (*F* = 0.136, *P* = 0.713).

#### P300

The results of repeated-measures ANOVAs show that the P300 amplitude (*F* = 0.511, *P* = 0.751) of the students in Task 1 is not significant when they pay attention to the social norms produced by different social distance groups. Therefore, the EEG amplitude caused by the social norms of different social distance groups in Task 2 is considered as the analysis object of the P300 component.

**FIGURE 4:**
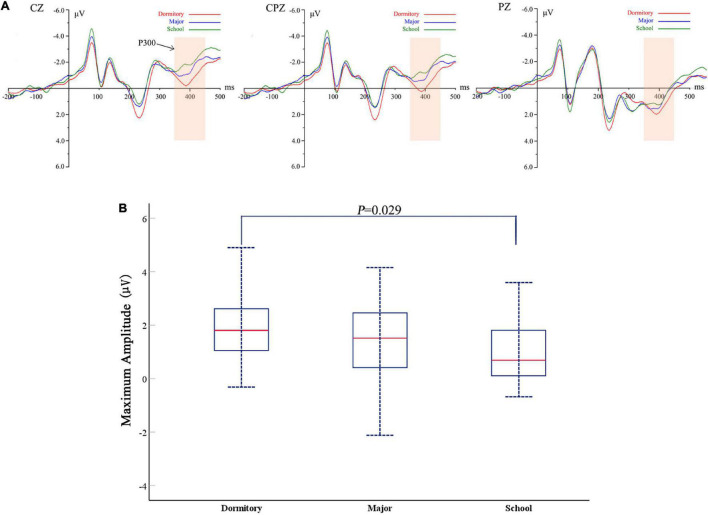
**(A)** Grand averaged ERPs waveforms at CZ, CPZ, and PZ of social distances in Task 2. **(B)** ANOVA results of social distances in Task 2.

We can see the average ERPs waveform induced by the three social distance groups in Task 2 at the CZ, CPZ, and PZ electrodes in Figure 4A. The ANOVA analysis results shown in Figure 4B confirmed that P300 at this stage was statistically significant (*F* = 3.690, *P* = 0.029). The EEG amplitude of CZ, CPZ, and PZ electrodes showed that the amplitude of the P300 component was larger in the group with closer social distance [dormitory (*M* = 1.920, SE = 1.343), major (*M* = 1.302, SE = 1.669), school (*M* = 0.959, SE = 1.090)]. In other words, people prefer to pay more attention to the attitudes and suggestions of the group closer to their society or spend more energy on the perceptual processing of the social norms of the group closer to their society.

### Influence of Social Norms on Willingness to Reuse Recycled Water

In order to validate the impact of social norms, the following two behavioral data were used to analyze whether social norms would provide reference conditions for college students’ willingness to recycle water.

**FIGURE 5:**
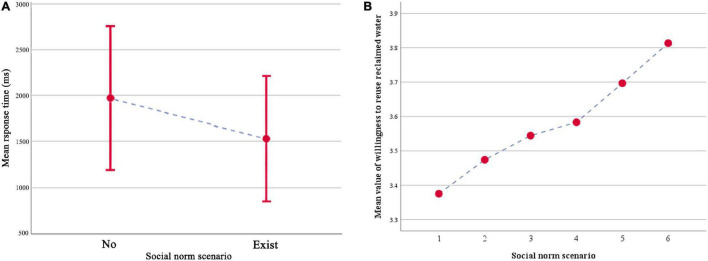
**(A)** The mean response time of Task 1 with no social norm and Task 2 with the social norm. Error bars depict the standard error of the mean. **(B)** Take the social norm value in Task 2 as a function of *a priori* category.

First, matched samples *T*-test were used to determine whether participants’ response time to keys could be shortened with social norms as reference. Table 3 shows the mean and standard deviation of the selection results and response time of participants’ intention to reuse recycled water in Task 1 and Task 2. The baseline key response time of participants was based on the key response time in Task 1, with an average level of 1974.737 ± 786.708 ms. The results are shown in Figure 5A demonstrate that the keystroke response time under the social norm reference condition was shorter than that without the social norm reference condition, and the difference between the two was statistically significant (*F* = 10.181, *P* < 0.001). The phenomenon of shorter decision response time suggested that social norms become the reference premise of decision execution in the process of the participants’ decision of their willingness to use recycled water. In other words, the introduction of the social norm as a guide reduced the amount of time and energy spent on the participants’ selection process.

**TABLE 3:** Mean and standard deviation (M ± SD) of willingness to reuse recycled water and response time in various stages of ERPs experiment.

The experimental stage		Willingness to reuse recycled water	Response time (ms)
Task 1		3.554 ± 1.601	1974.737 ± 786.708
Task 2	High social norm scenarios	4.110 ± 1.662	1503.870 ± 1184.340
	Low social norm scenarios	3.160 ± 1.595	1556.190 ± 1238.536

Secondly, a one-way ANOVA was used to verify that the participants’ willingness to reuse recycled water was indeed different under the influence of different social norms (*F* = 12.067, *P* < 0.001). The social norm scenarios in Task 2 were divided into Low and High Scenarios based on the mean intentions to reuse recycled water collected by Task 1 (Mean = 3.554, SD = 1.601). The participants’ willingness to reuse recycled water under the influence of high social norms scenarios (Mean = 4.110, SD = 1.662) was higher than the previous baseline willingness, while the participants’ willingness to use recycled water under the influence of low social norms scenarios (Mean = 3.160, SD = 1.595) was reduced (Table 3). Furthermore, Figure 5B shows the changes in the participants’ recycled water reuse intention under the influence of different social norm values. The increase of the participants’ willingness to reuse recycled water along with social norm value suggested an obvious correlation between both factors. One-way ANOVA was conducted with the willingness to reuse recycled water as the dependent variable and the social norm scenario type as the factor. The results also showed that the social norm scenario type had a significant impact on the participants’ willingness to use recycled water (*F* = 688.433, *P* < 0.001).

## Discussion

### Discussion of Findings

For the purpose of the research, two phases of experiments were carried out. Firstly, in the social norm activation scenario experiment, the results have shown that the social norms after activation affected the college students’ willingness to use recycled water. The results also suggested that descriptive social norms can lead to a greater degree of willingness to accept recycled water. The ERP experimental methods were adopted in the second stage of the experiment. In order to explore the social norm information processing process of college students for different social groups in the situation of recycled water reuse, a variant of the classic “Judge-Advisor System” paradigm was carried out. Through observation of FRN and P300 components in different experimental control groups, they reflected the motivational force of social norms developed by different social distances on college students’ willingness to use recycled water. The results have shown that the activation level of the FRN component in the context of social norm deviation was higher than that in the context of no social norm deviation, and positive deviation could activate larger FRN amplitude in the experiment. The results of the P300 component analysis have shown that college students who participated in the experiment had a larger P300 amplitude to the social norms consistent with their willingness, meaning that students allocated more attention resources. In addition, this study also observed the influence of social distance on college students’ acceptance of social norms. The results have shown that when college students deviated from the social norms proposed by the group with closer social distance, the greater the FRN amplitude was. Moreover, when facing the social norms proposed by the same dormitory group, the FRN amplitude caused by the positive deviation was larger. However, this conclusion has not been confirmed in the same major group and the same school group. The P300 component analysis results showed that college students activated a larger P300 component when they saw the suggestions of social norms promoted by groups closer to their society.

For the condition that one’s own opinions were consistent with social norms, the deviation between them induced a relatively large FRN amplitude, which was related to the cognitive processing after paying attention to the group judgment. That shows that when participants discovered that their willingness was inconsistent with the given social norms, they produced incorrect perceptual signals, resulting in strong negative emotions or motives. It may be one of the incentives for social norms to intervene in such behavioral decisions. At the same time, higher FRN amplitude was detected when the group social norm was higher than the participant’s social norm and when individual willingness and social norm were deflected. This suggested that during behavioral intention selection of using recycled water, the group whose own intention is lower than the general environment will make participants have a more significant sense of error. On the contrary, when the individual’s willingness to reuse recycled water was higher than the social norms proposed by other groups, the participants’ sense of error was comparatively low. Combined with the observation of behavioral data, the low social norms had no significant impact on the participants’ final decision of willingness to use recycled water. It is possible that the inducement for this phenomenon was that using recycle water protects the environment and provided participants a pleasant emotional valence. This emotion caused them to have an implicit attitude of “it is right for me to have a high willingness to use recycled water,” and ultimately help reduce the guilt of breaking low social norms. Therefore, in the actual promotion process of recycled water, we should establish a high moral appraisal of recycled water usage behavior at the individual perception level through environmental motivation. In the scenario of recycled water usage, we attempted to induce individuals’ positive psychology of upward social comparison to alleviate the negative influence of others’ negative willingness of recycled water usage on college students’ behavior choices. Although the inconsistency between individual and group attitudes might cause participants to feel as if they were making mistakes, the results of P300 showed that participants tended to devote attention resources to the suggestions of social norms that were consistent with their willingness. People usually recognize and praise people who have the same attitude as themselves. Therefore, our findings could suggest that the P300 component is related to the evaluation process of self-benefit, which is consistent with the view proposed by Chen et al. (2017). Self-efficacy here largely describes the quality of others’ self-social evaluation. When combined with behavioral decision-making data, although college students are more likely to pay more attention when social norms are consistent with their willingness to use recycled water, violating social expectations may cause more intense conflict. Under this conflict, college students were more inclined to make willing behavioral judgments that fit the requirements of social norms in the next step.

In addition, this study also observed that when individual willingness differed from social norms, the social distance was found to be one of the important factors affecting college students’ perception of social norms. The FRN indicated that when the individual willingness deviated from the social norm, the closer the social distance was and the larger the FRN amplitude was. This conclusion was especially obvious in the same dormitory group. This phenomenon was interpreted as that driven by self-expansion, the closer others will be more easily integrated into the self-structure to form self-friend overlapping (Liu et al., 2021). Therefore, when the behavior intention is inconsistent with the group with a similar self-structure, there would be a tendency to have more false conflict perception. The ability to utilize the conflict perception will be a breakthrough point to improve the willingness of college students to use recycled water. The result suggested that the positive deflection would cause larger FRN amplitude only in the same dormitory group, but the main effect analysis was not significant in the same major group and the same school group. Presumably, because only dormitory mates were visualized in the “friends” group of participants, while there were no differences between the major group and school group and the “strangers” group.

The P300 observed in Task 2 indicates the participants’ motivation and encoding of the emotional meaning of the social norms created by these three social distance groups. The social norms are given by people living in the same dormitory induced the highest P300 amplitude, followed by the same professional group, and the lowest among people living in the same school. It suggested that the use of recycled water promoted by close people drew higher attention from college students. Existing studies have shown that when suggestions deviate from their own were given by strangers, participants would get a lower P300 amplitude when they accept the group norms of the same strangers again (Schnuerch et al., 2014). In other words, the deviations from social norms tend to cause participants to pay less attention to the social norms of the same group. Surprisingly, this phenomenon was not observed in this study. It is probably because compared to strangers, groups with closer social distance have more social bonds which minimize the likelihood of ignoring social norms caused by conflict.

In sum, experimental results have shown that the impact of social norms on participating college students’ willingness to use recycled water was not only related to the degree of deviation between individual will and social norms but also closely related to the group presenting social norms. When confronted with a situation involving social norms, participants tend to make a decision that is close to the norm. When they deviated from social norms, participants subconsciously believed that they had made a mistake. At the same time, people also pay more attention to the information of social norms presented by intimate groups. The advice and practice of “friends” greatly affected the willingness of the participating individual college students to use recycled water. Therefore, social norms would be an influential aspect that cannot be ignored in the promotion of recycled water on campus.

### Limitations and Future Research Directions

It is important to note that this study still has some limitations. On one hand, this study demonstrates the possibility of the cognitive impact of short-term social norms on recycled water usage of college students from a neural level. However, in reality, social norms have a long-lasting and subtle influence on individual behavior. To pay attention to the long-term impact of social norms on college students’ willingness to use recycled water is something we can further study in the future. On the other hand, this study focuses on the implementation of recycled water on campus from the perspective of the social norm. However, it is unclear whether social norms can have the same motivational effect on other groups with more complex demographics. In future studies, more groups with diverse characteristics will be found to explore the impact of social norms on their willingness to use recycled water.

## Conclusion

The public’s resistance to the reuse of recycled water has become a constraint to further promote the use of recycled water as an alternative water source. However, at present, the price of tap water is too low to encourage the public to reuse recycled water by widening the price difference between them. Therefore, this study is expected to find ways to improve college students’ willingness to reuse recycled water from the perspective of social norms.

Conclusions of the study are as follows: (1) When the participants are guided by the suggestions of social norms, they will tend to make behavioral decisions consistent with the social norms, and at the same time shorten the keystroke response time. (2) When individual willingness is inconsistent with social norms, it will cause greater FRN amplitude and smaller P300 amplitude, while when they are consistent, it will cause smaller FRN amplitude and larger P300 amplitude. (3) The social norm deviation scenario caused by the group with closer social distance will cause greater FRN and P300 amplitude. When forming the social norms of recycled water usage among college students, we should begin with the group with close social relations, and then form the social norms of active use of recycled water from a point to an area. The upward social comparison psychology and group identity will support and superimpose each other, resulting in a favorable atmosphere of the use of recycled water in a virtuous circle and to achieve the expected goal of promoting college students’ willingness to use recycled water through social norms.

## Data Availability Statement

The raw data supporting the conclusions of this article will be made available by the authors, without undue reservation.

## Ethics Statement

The studies involving human participants were reviewed and approved by the Internal Review Board of the Laboratory of Neuromanagement in Engineering, Xi’an University of Architecture and Technology. The patients/participants provided their written informed consent to participate in this study.

## Author Contributions

XL: responsible for the conceptualization, supervision, and funding acquisition. SC: responsible for the conceptualization, data curation, formal analysis, software, investigation, writing – original draft, and validation. XG: responsible for the methodology and revising the manuscript critically for important intellectual content. HF: responsible for the methodology, project administration, resources, funding acquisition, and validation. All authors contributed to the article and approved the submitted version.

## Conflict of Interest

The authors declare that the research was conducted in the absence of any commercial or financial relationships that could be construed as a potential conflict of interest.

## Publisher’s Note

All claims expressed in this article are solely those of the authors and do not necessarily represent those of their affiliated organizations, or those of the publisher, the editors and the reviewers. Any product that may be evaluated in this article, or claim that may be made by its manufacturer, is not guaranteed or endorsed by the publisher.

## Appendix A

**TABLE A1:** Symbol table.

Denotation	Abbreviation
event-related potentials	ERPs
anterior insula	AI
anterior cingulate cortex	ACC
feedback-related negativity	FRN
Judge-Advisor System	JAS
