# Supplementary figures and images for: Can Social Norms Promote Recycled Water Use on Campus? The Evidence From Event-Related Potentials
Source: Front Psychol. 2022 Feb 4;13:818292. doi: 10.3389/fpsyg.2022.818292 (PMC8856723; doi:10.3389/fpsyg.2022.818292)

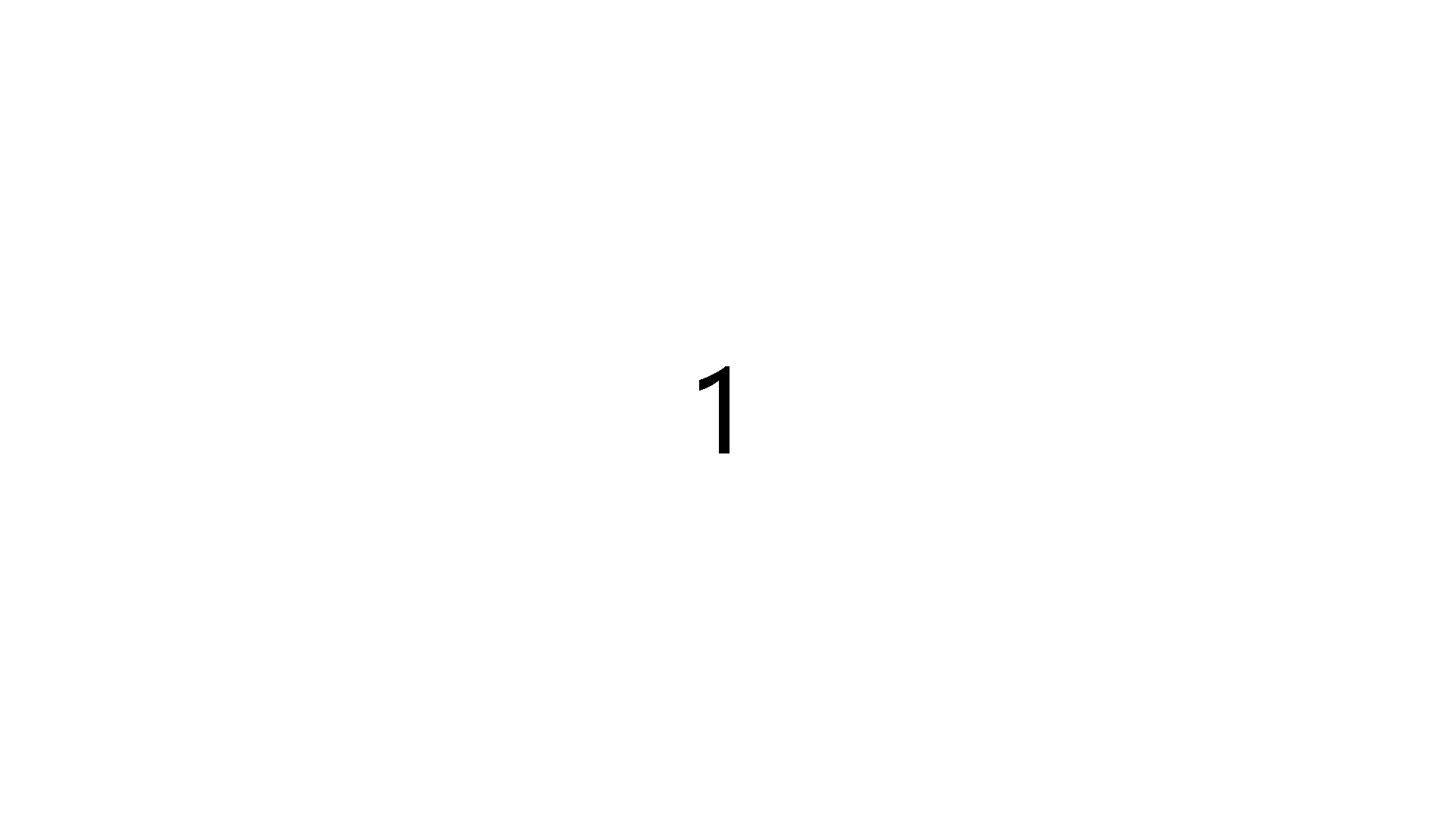

Supplement: Supplementary file 1 [file Data_Sheet_1.ZIP › Stimulus/1.jpg]

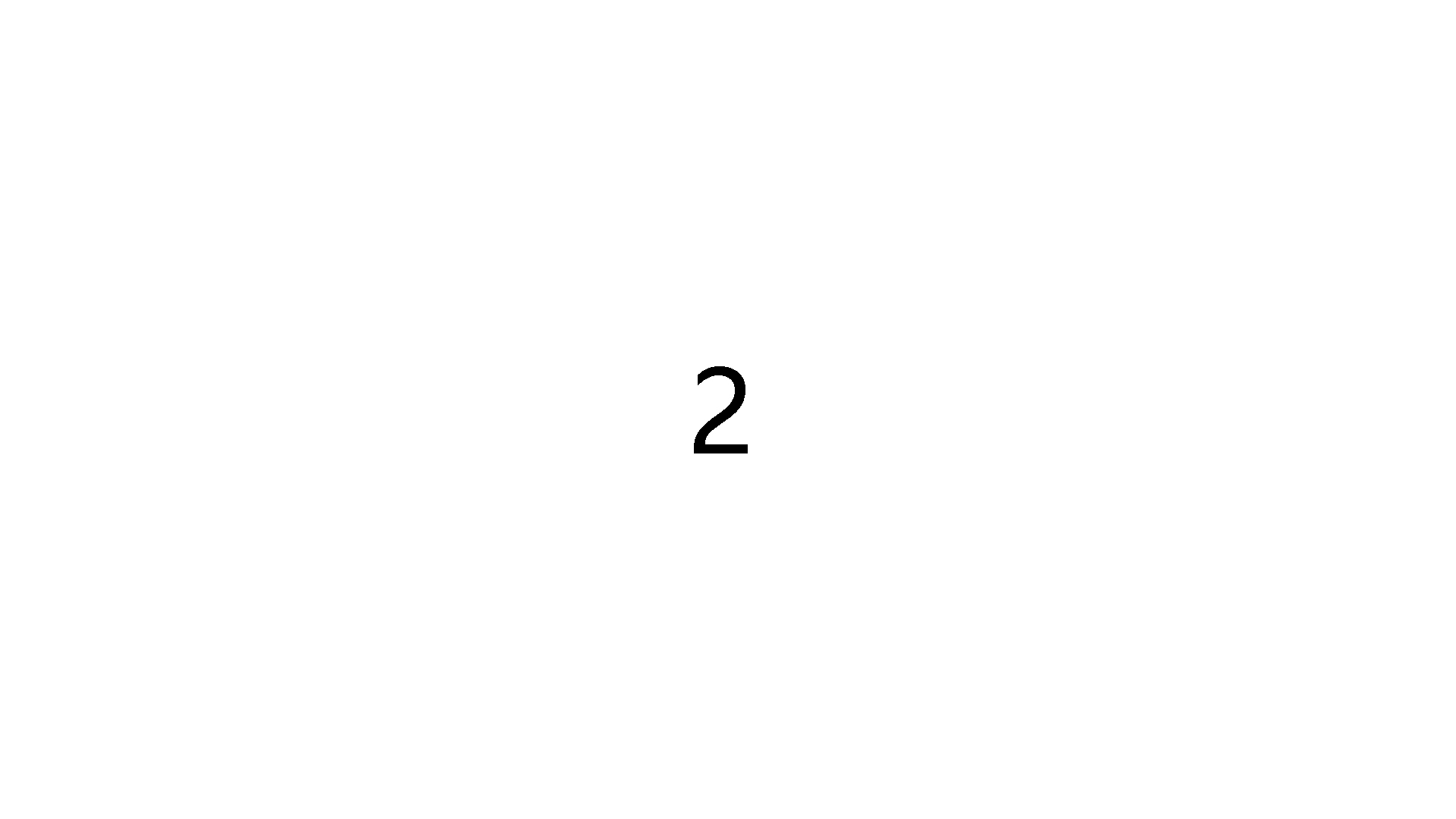

Supplement: Supplementary file 1 [file Data_Sheet_1.ZIP › Stimulus/2.jpg]

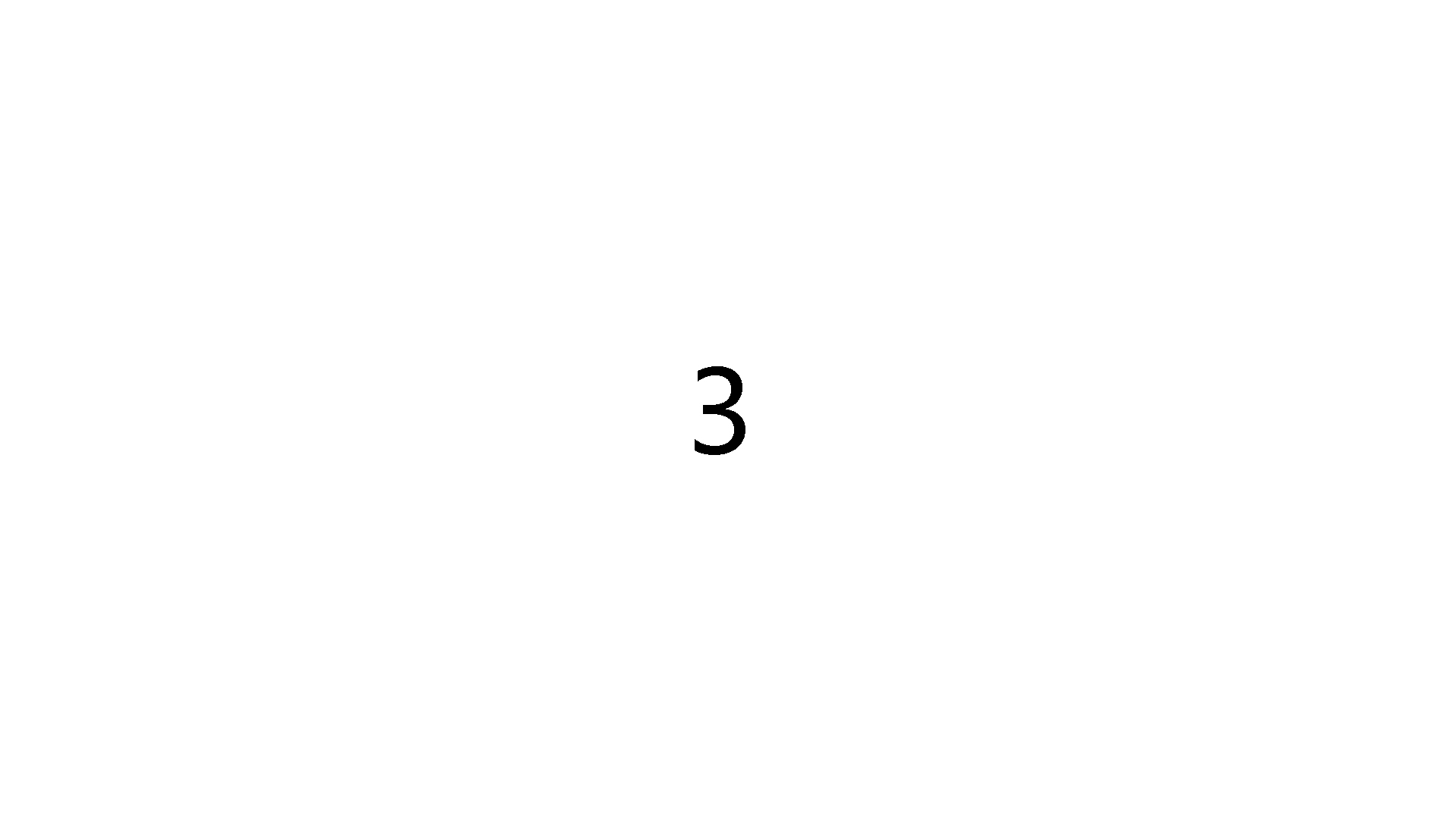

Supplement: Supplementary file 1 [file Data_Sheet_1.ZIP › Stimulus/3.jpg]

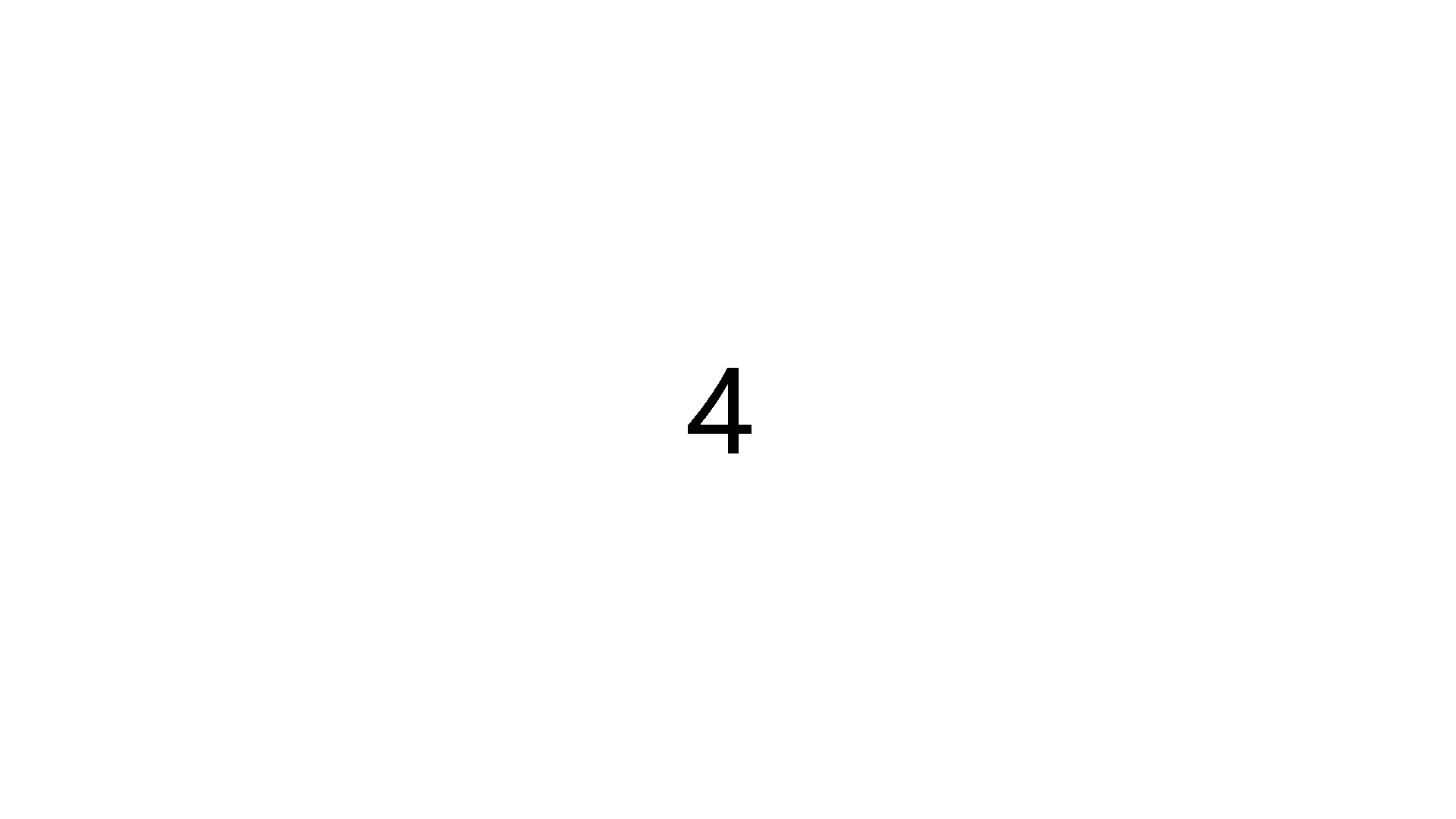

Supplement: Supplementary file 1 [file Data_Sheet_1.ZIP › Stimulus/4.jpg]

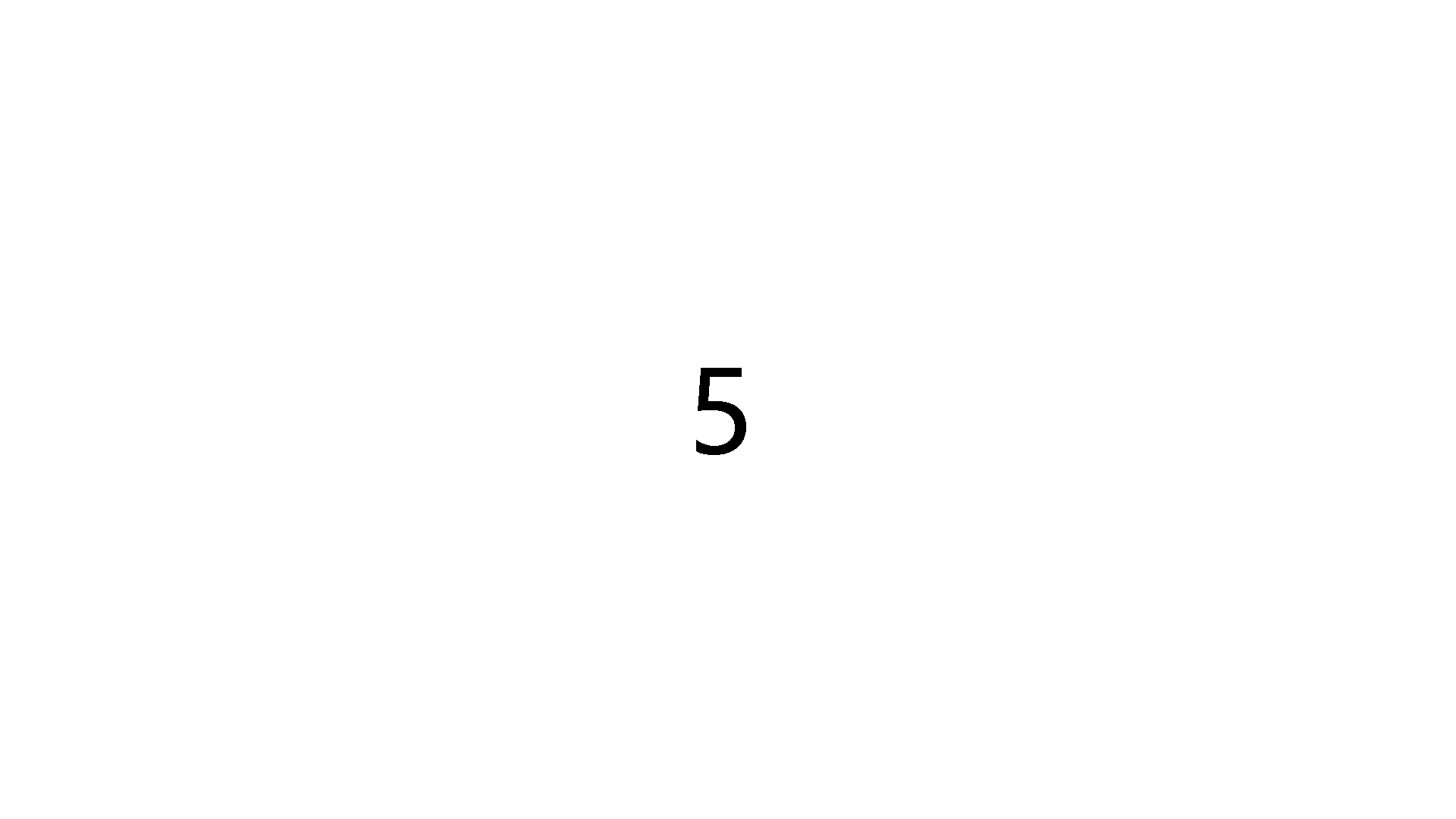

Supplement: Supplementary file 1 [file Data_Sheet_1.ZIP › Stimulus/5.jpg]

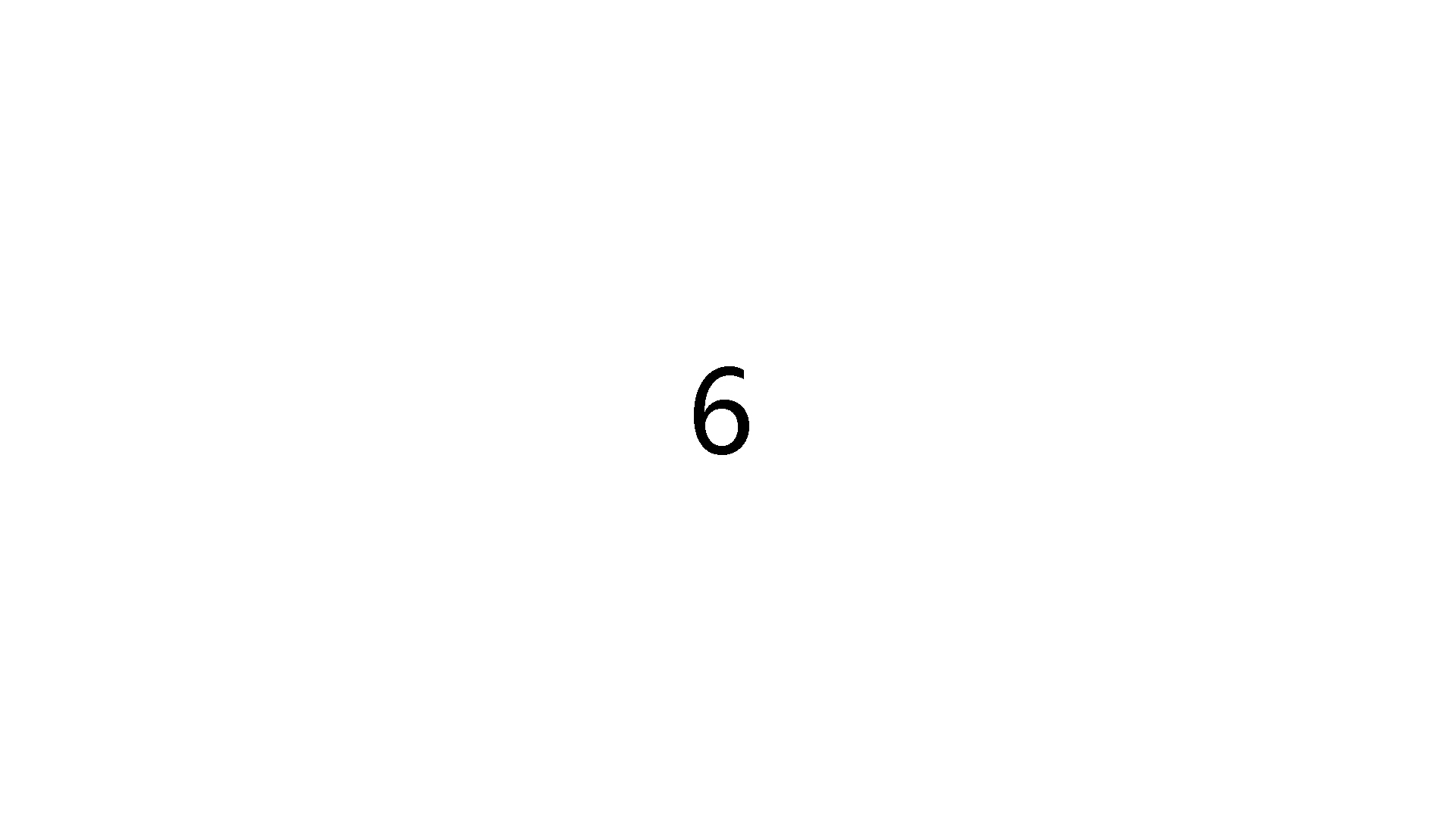

Supplement: Supplementary file 1 [file Data_Sheet_1.ZIP › Stimulus/6.jpg]

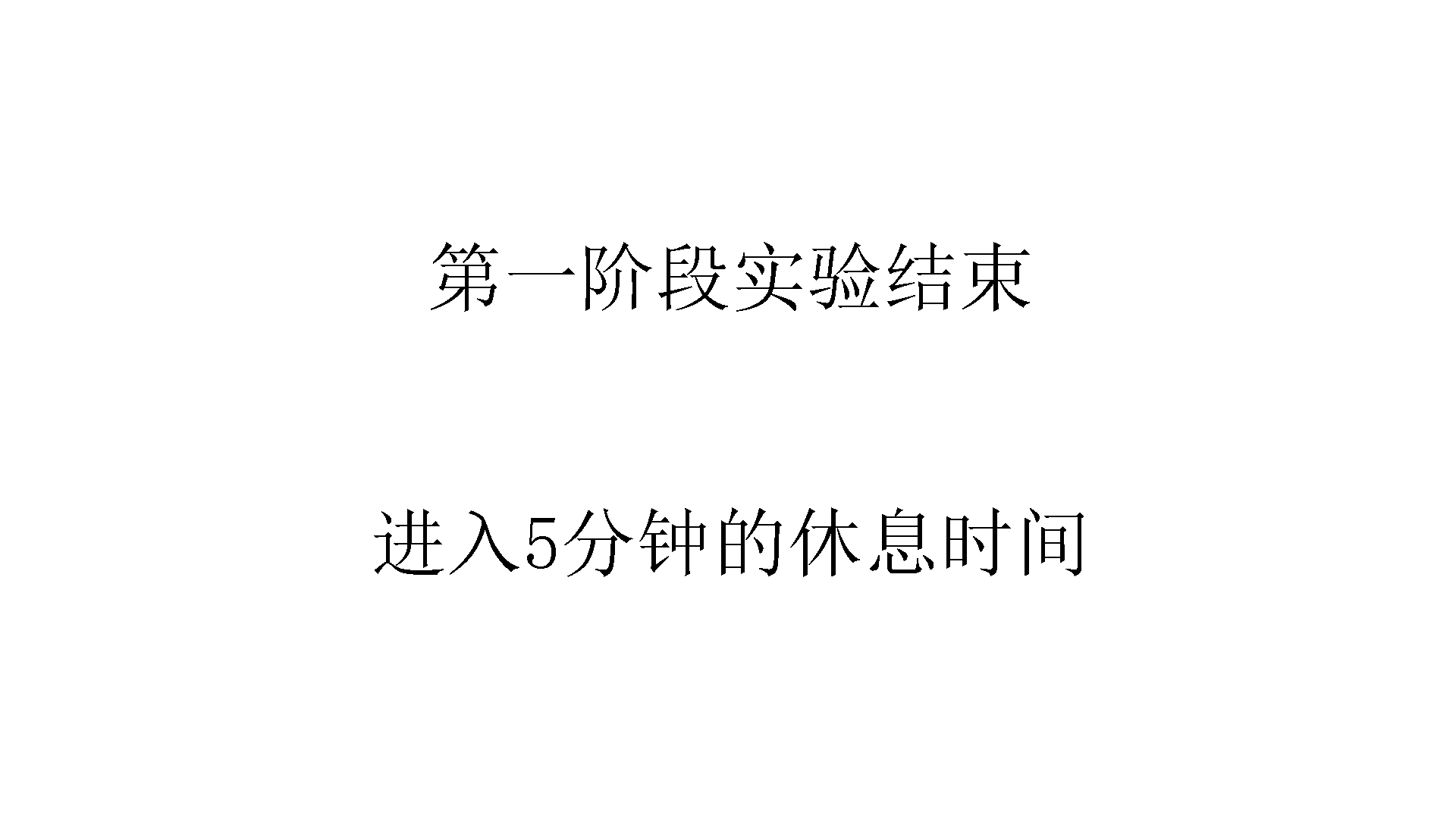

Supplement: Supplementary file 1 [file Data_Sheet_1.ZIP › Stimulus/break.jpg]

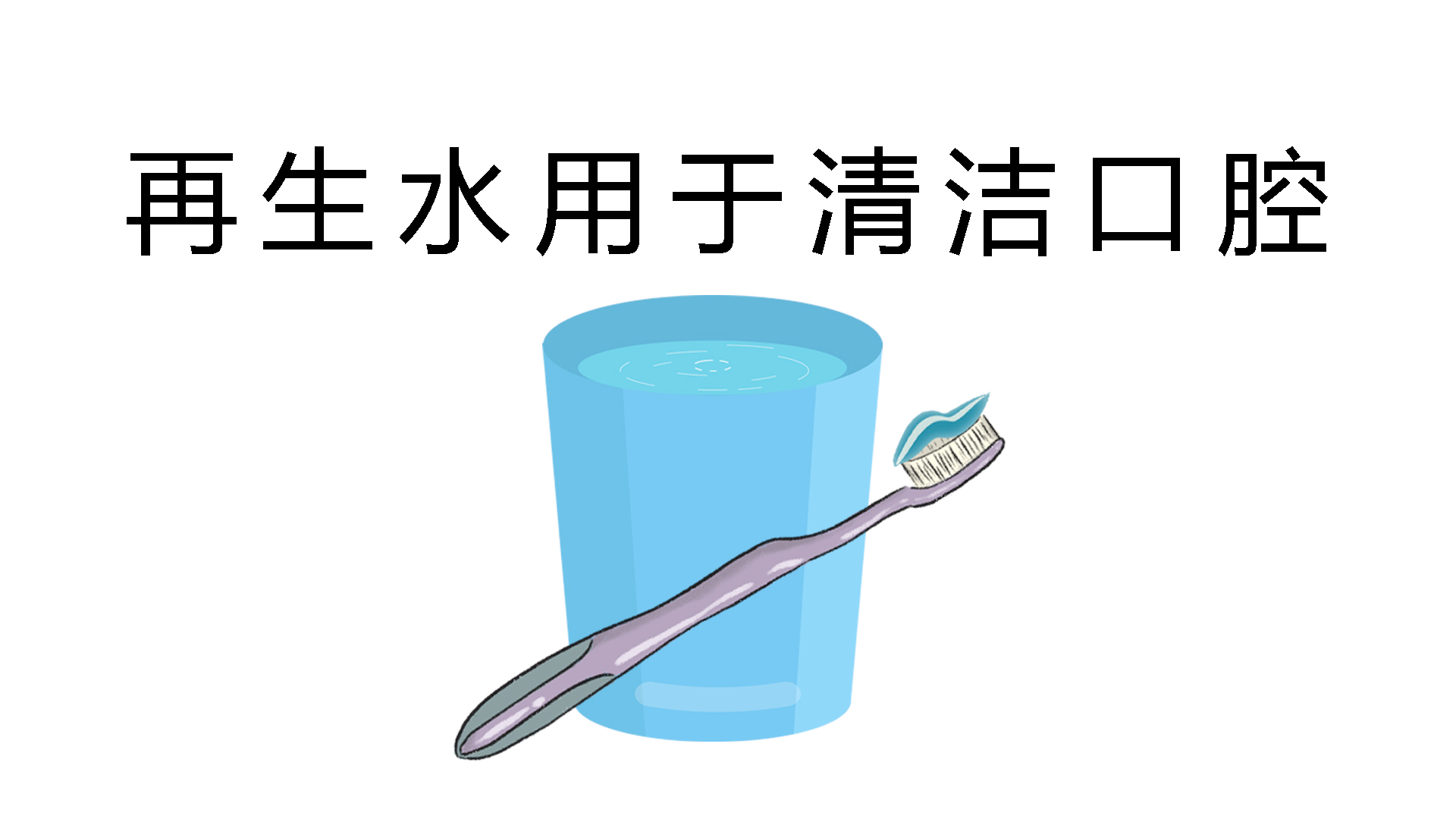

Supplement: Supplementary file 1 [file Data_Sheet_1.ZIP › Stimulus/brushing.jpg]

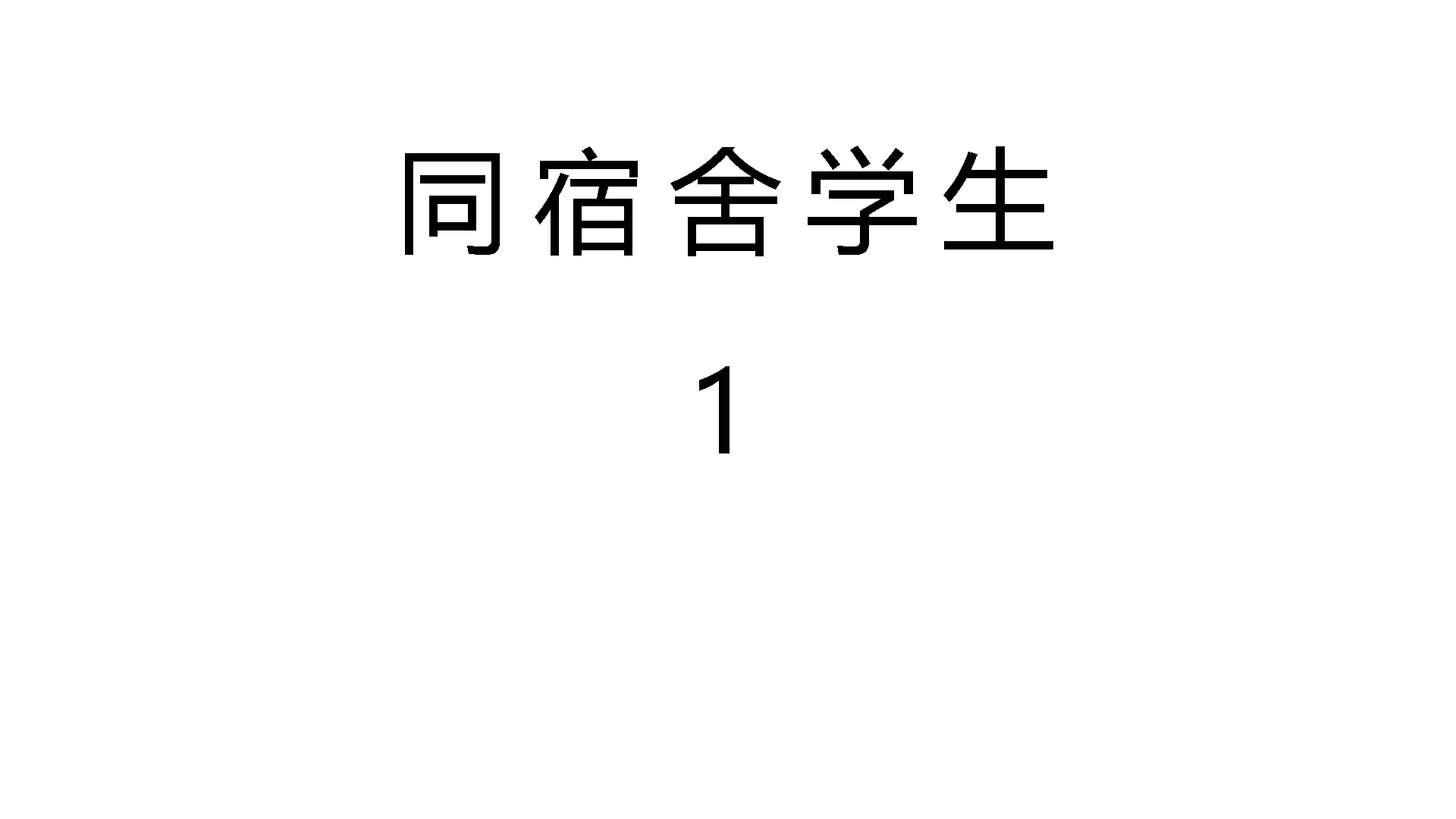

Supplement: Supplementary file 1 [file Data_Sheet_1.ZIP › Stimulus/dormitory1.jpg]

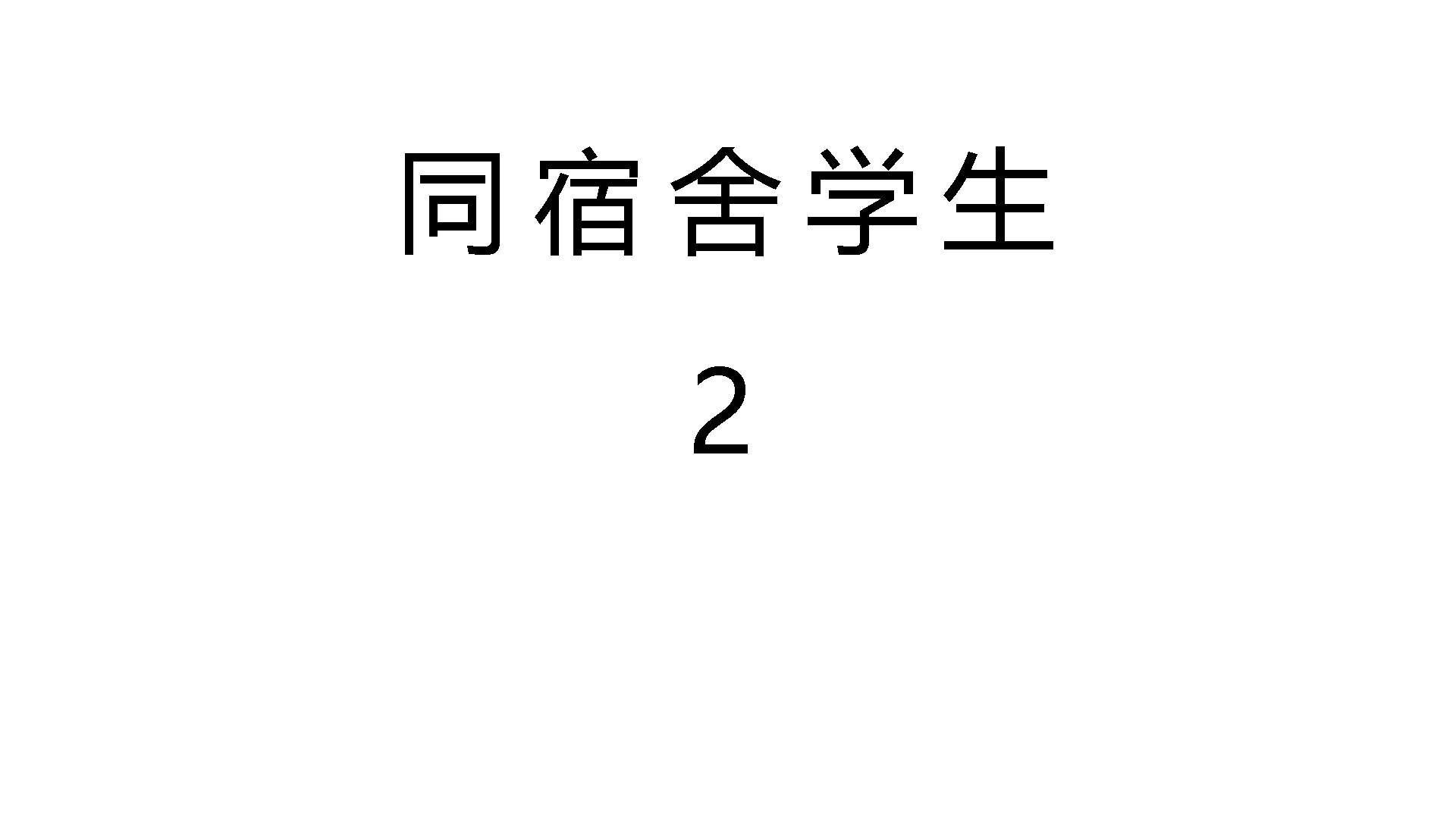

Supplement: Supplementary file 1 [file Data_Sheet_1.ZIP › Stimulus/dormitory2.jpg]

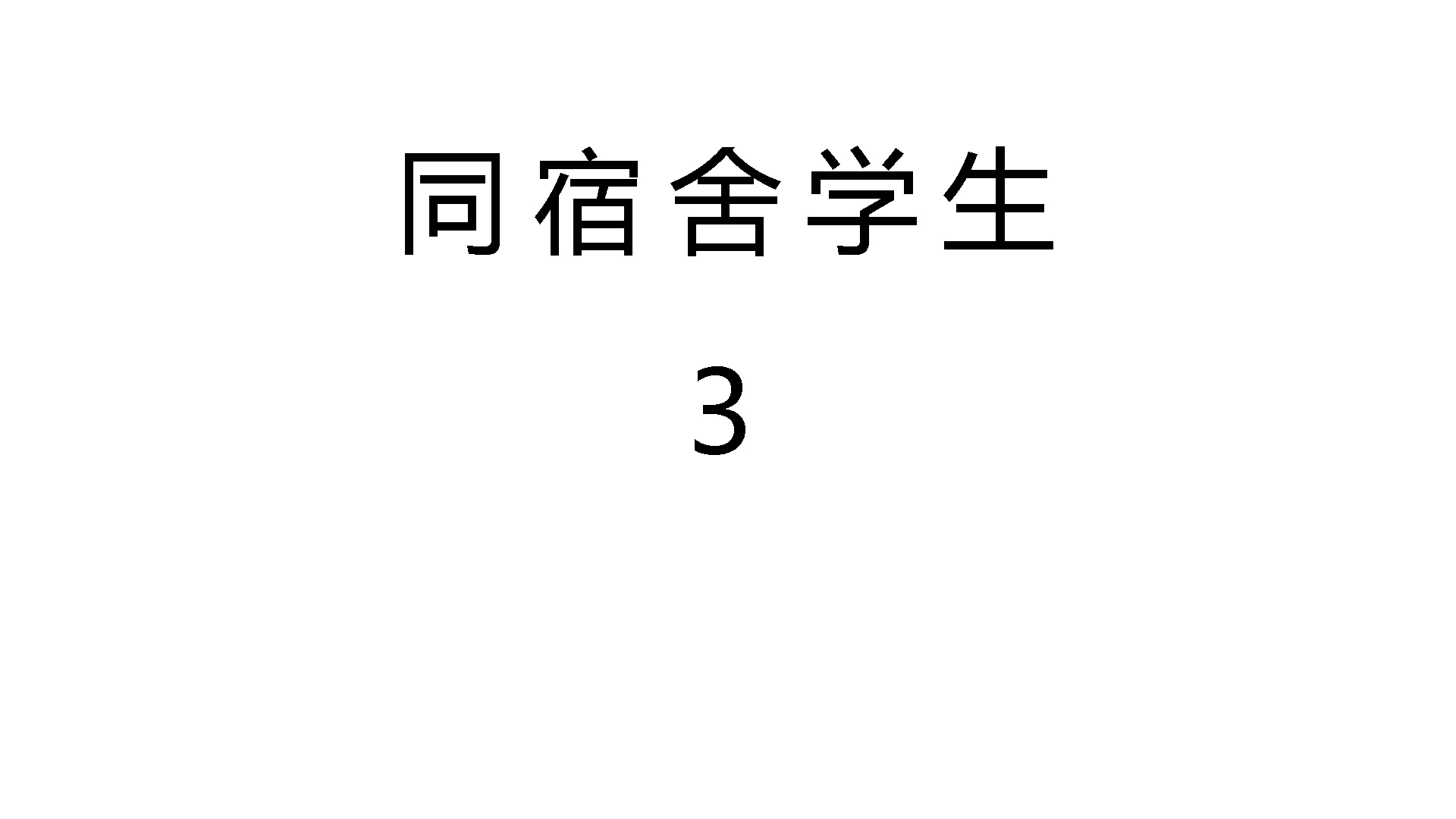

Supplement: Supplementary file 1 [file Data_Sheet_1.ZIP › Stimulus/dormitory3.jpg]

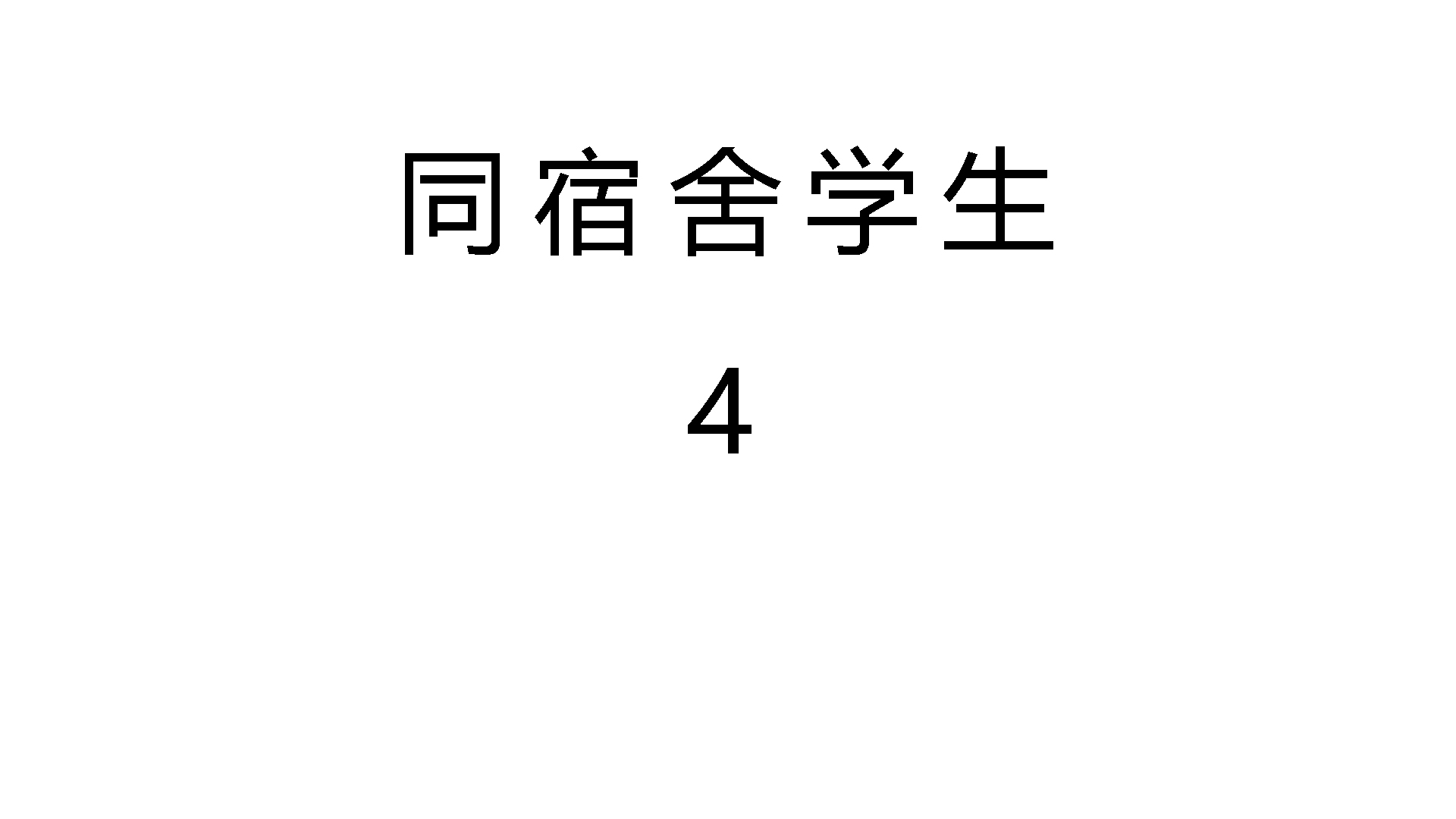

Supplement: Supplementary file 1 [file Data_Sheet_1.ZIP › Stimulus/dormitory4.jpg]

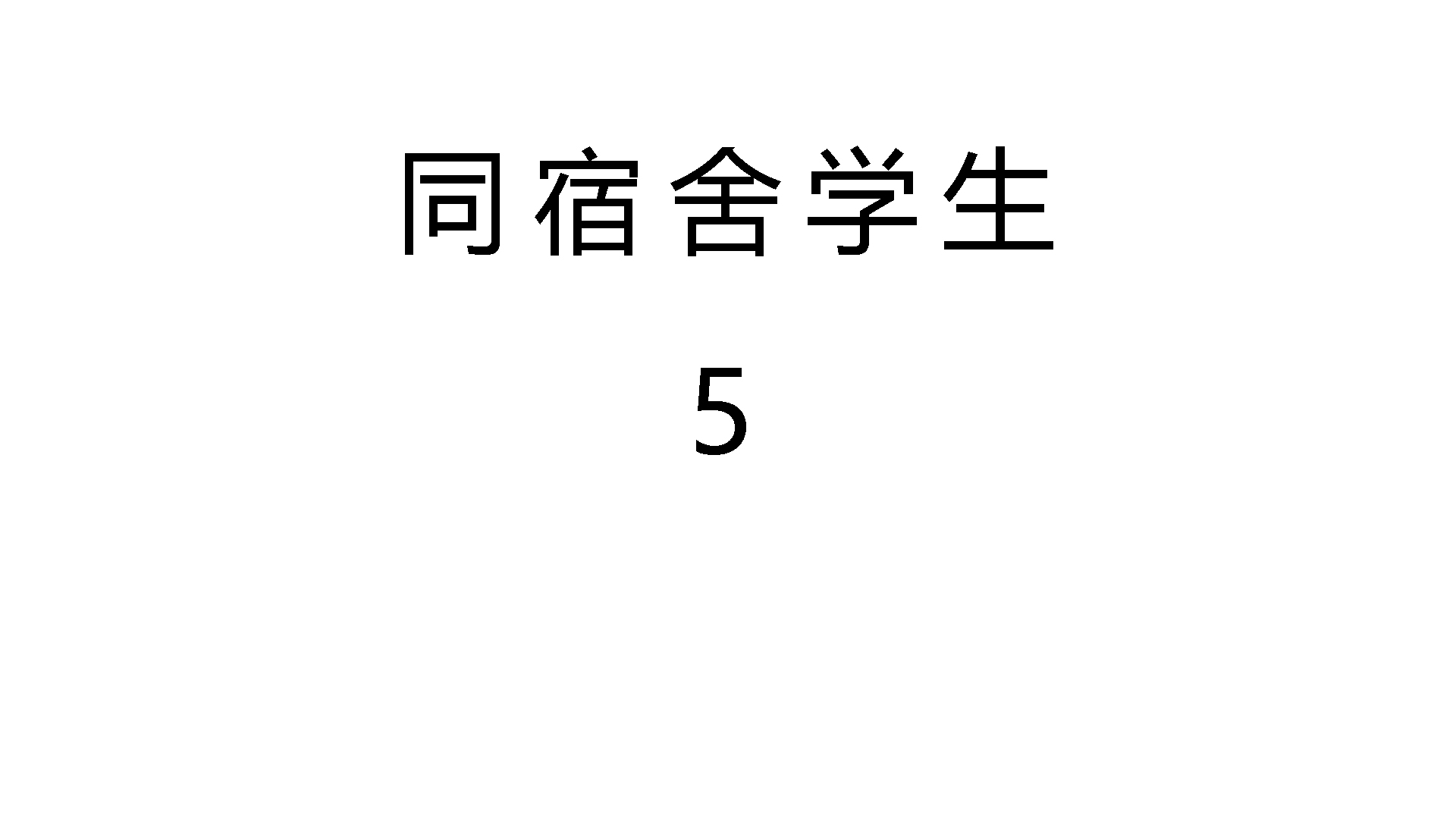

Supplement: Supplementary file 1 [file Data_Sheet_1.ZIP › Stimulus/dormitory5.jpg]

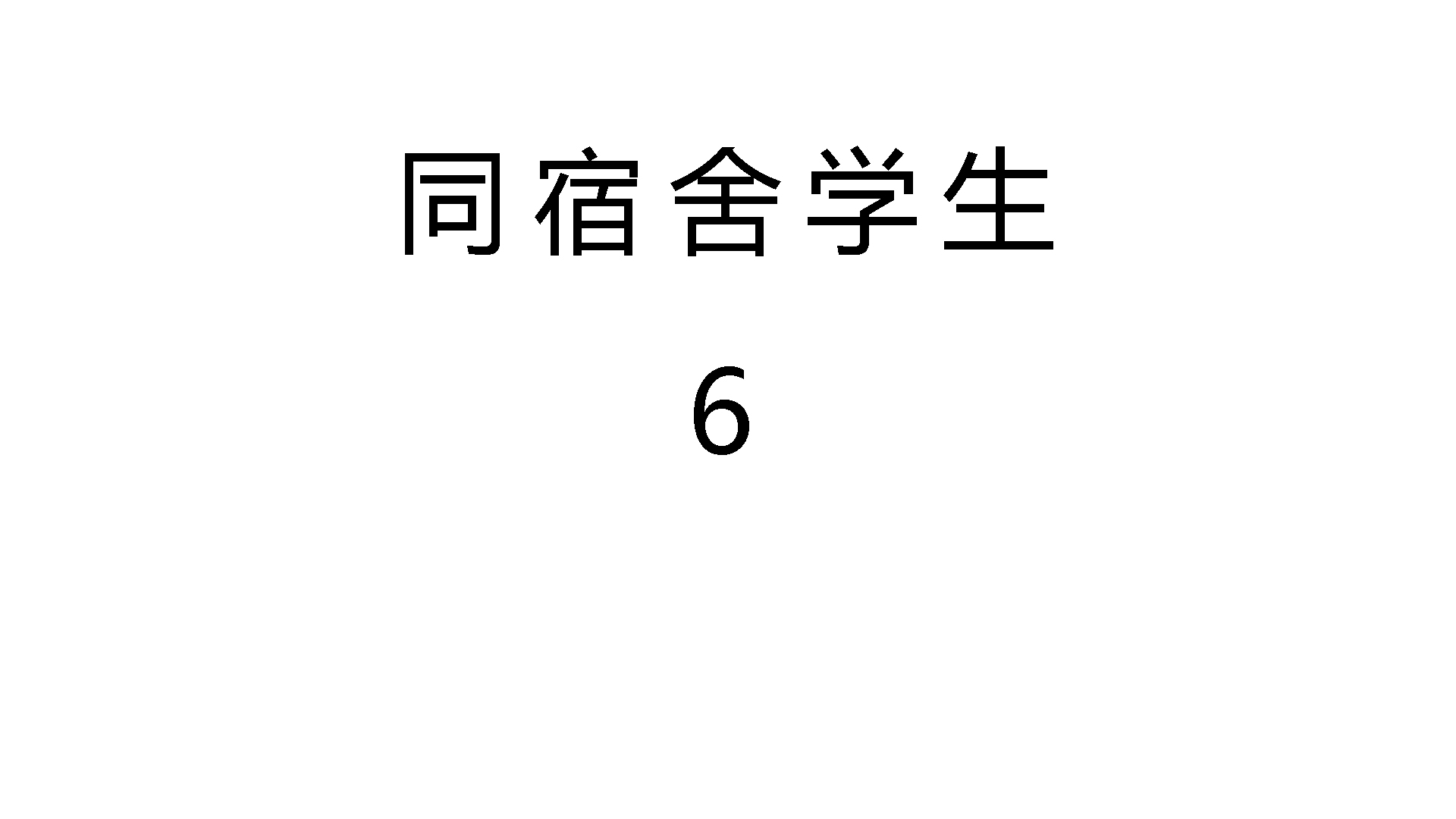

Supplement: Supplementary file 1 [file Data_Sheet_1.ZIP › Stimulus/dormitory6.jpg]

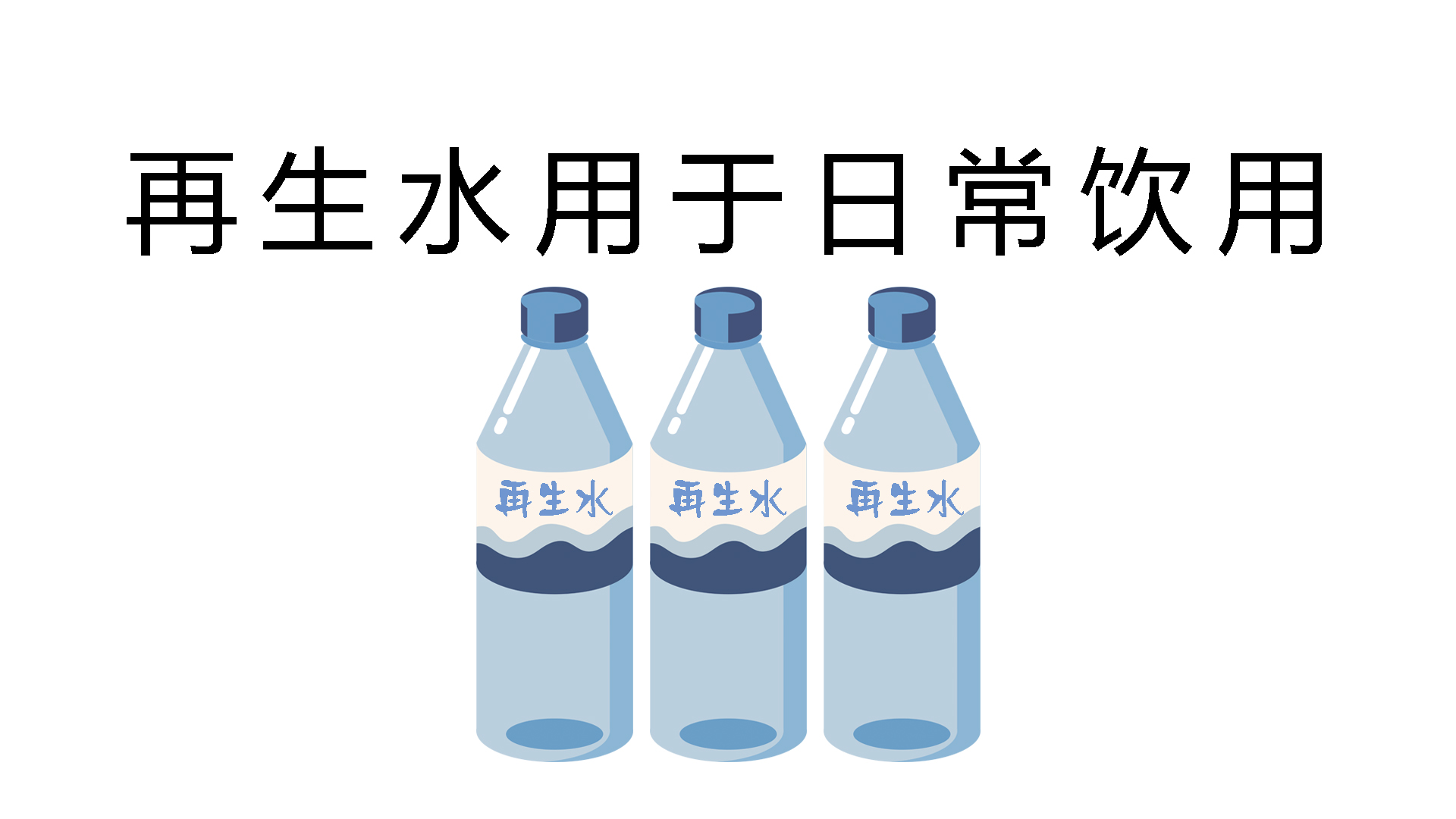

Supplement: Supplementary file 1 [file Data_Sheet_1.ZIP › Stimulus/drinking.jpg]

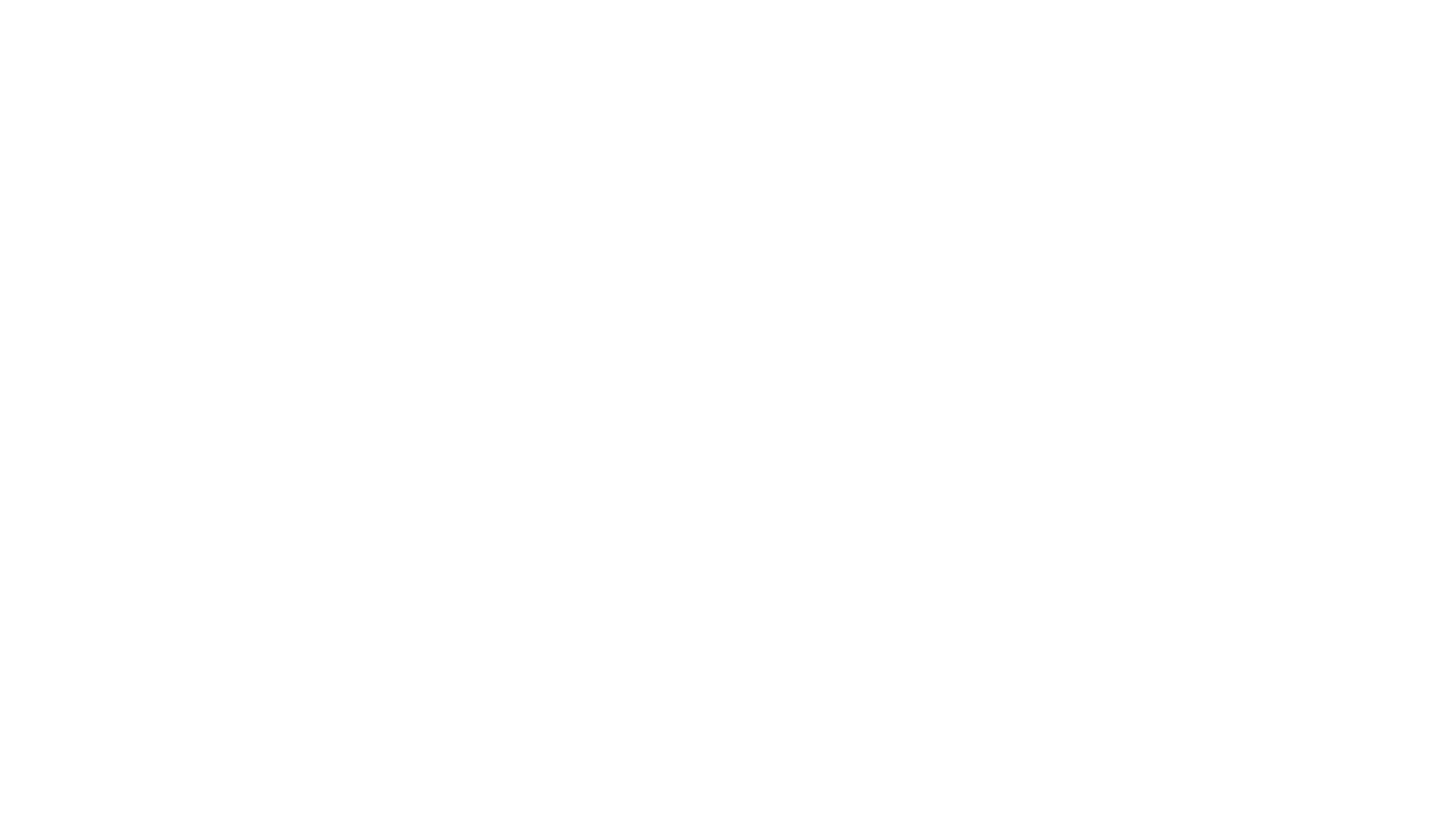

Supplement: Supplementary file 1 [file Data_Sheet_1.ZIP › Stimulus/Empty.jpg]

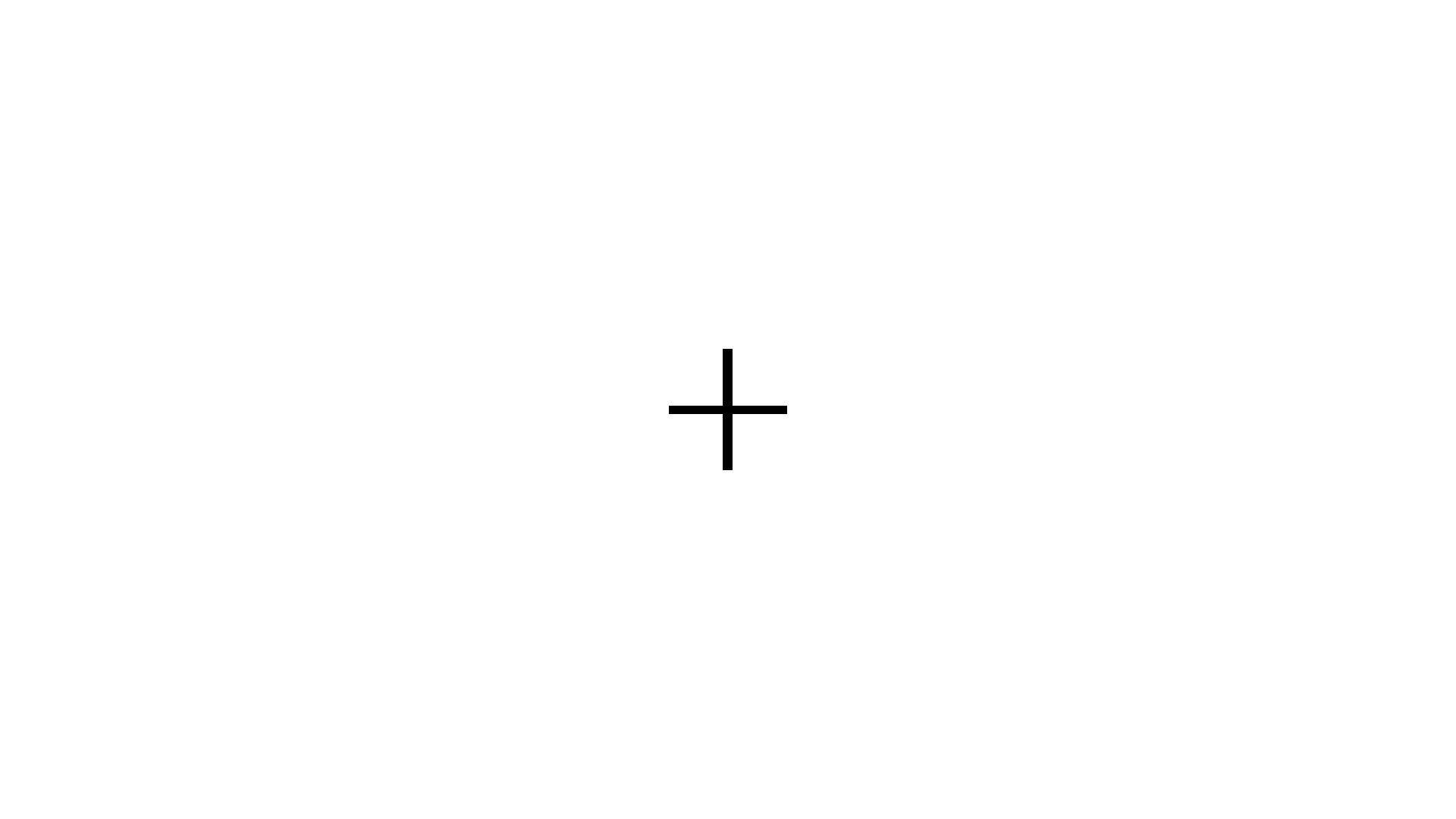

Supplement: Supplementary file 1 [file Data_Sheet_1.ZIP › Stimulus/focus.jpg]

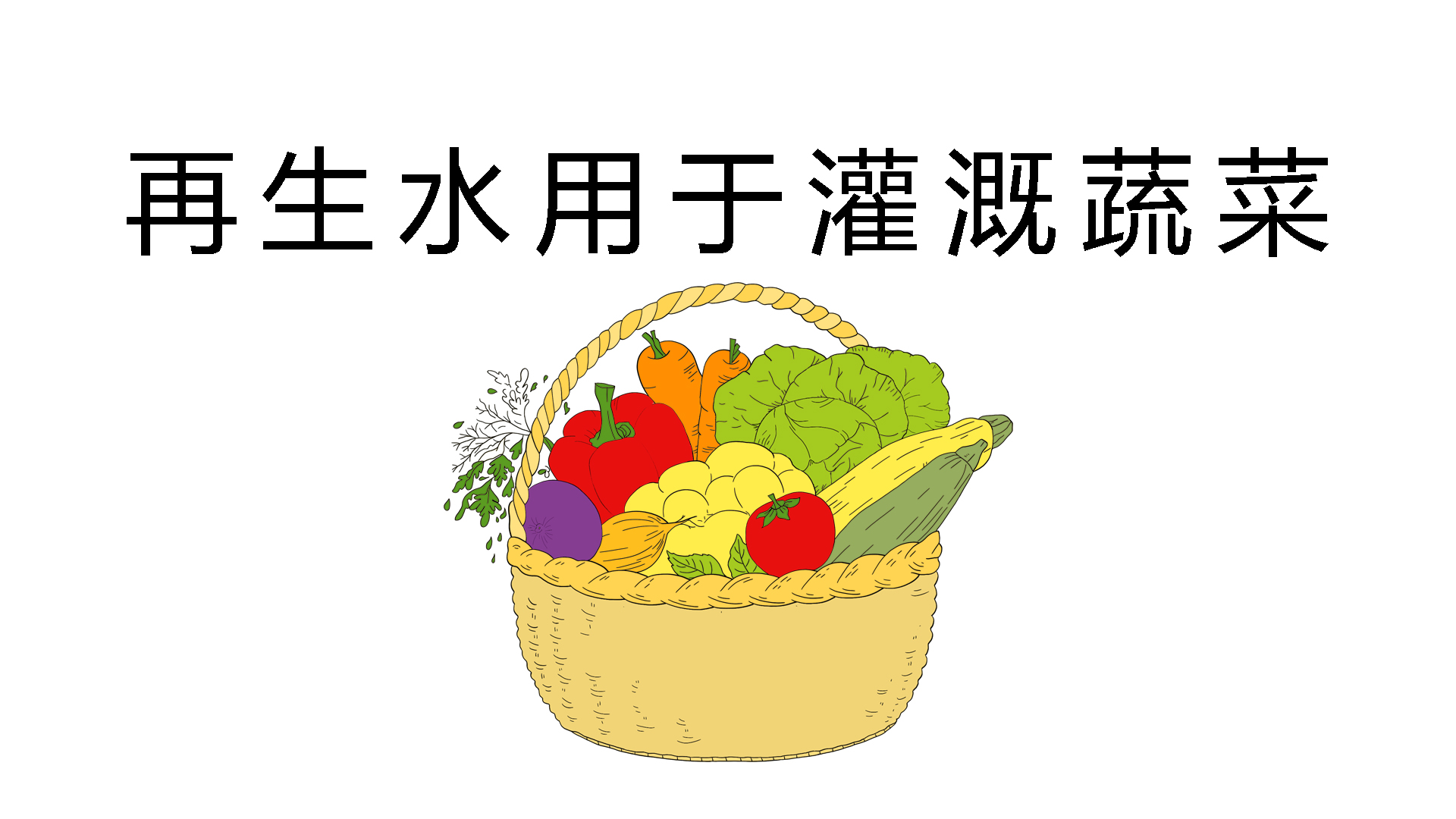

Supplement: Supplementary file 1 [file Data_Sheet_1.ZIP › Stimulus/IrrigationOfVegetables.jpg]

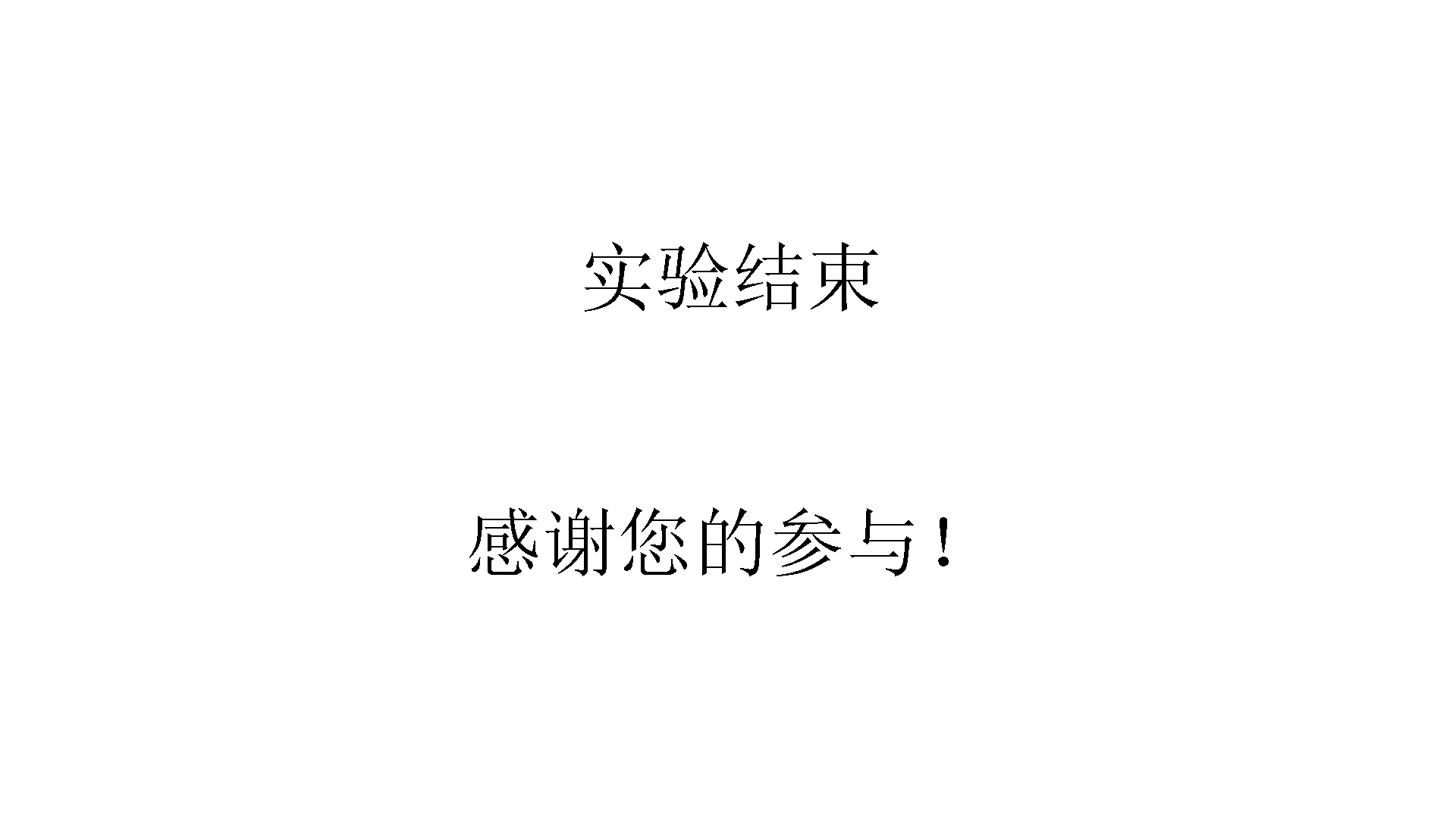

Supplement: Supplementary file 1 [file Data_Sheet_1.ZIP › Stimulus/jieshuyu.jpg]

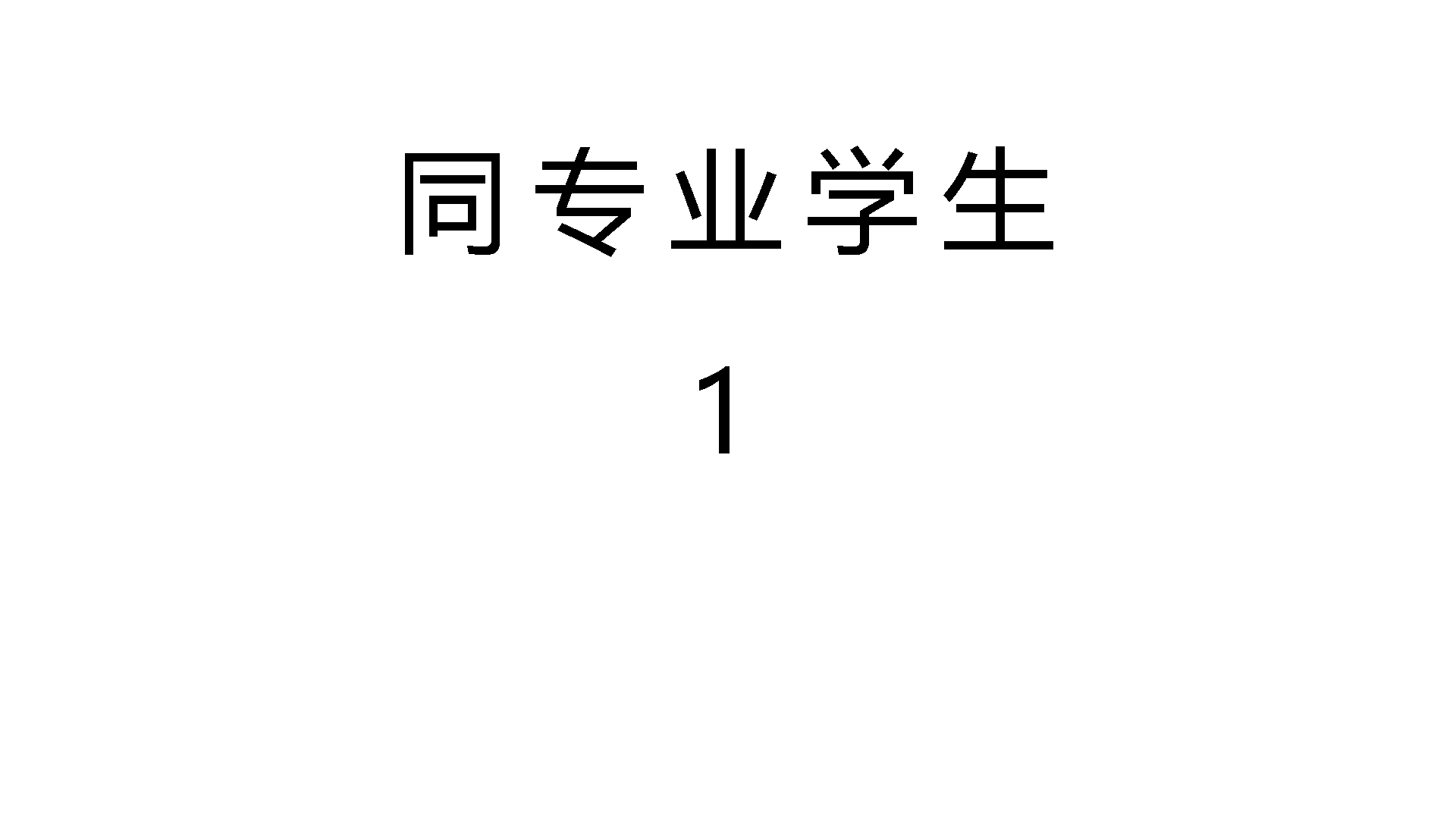

Supplement: Supplementary file 1 [file Data_Sheet_1.ZIP › Stimulus/major1.jpg]

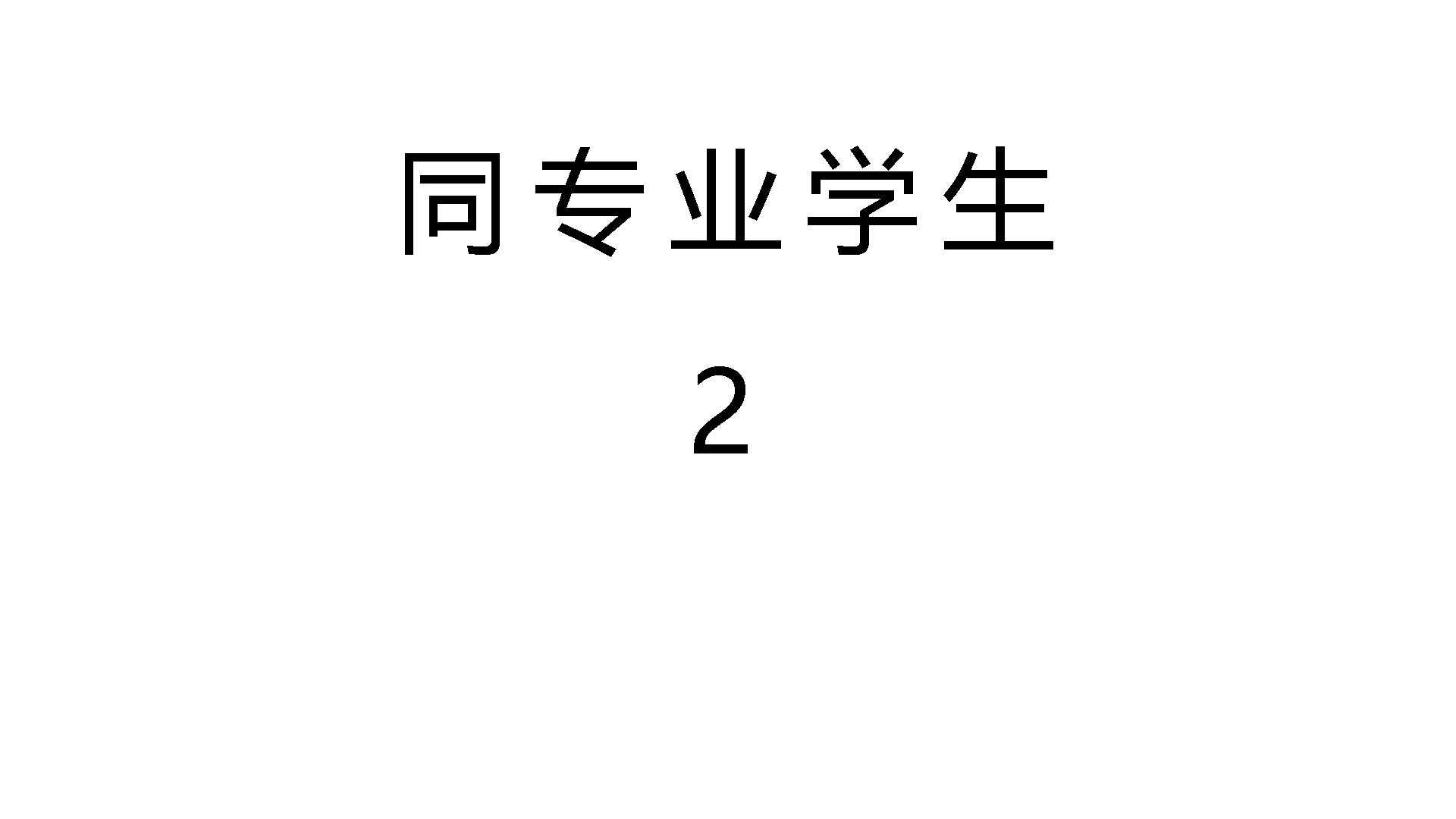

Supplement: Supplementary file 1 [file Data_Sheet_1.ZIP › Stimulus/major2.jpg]

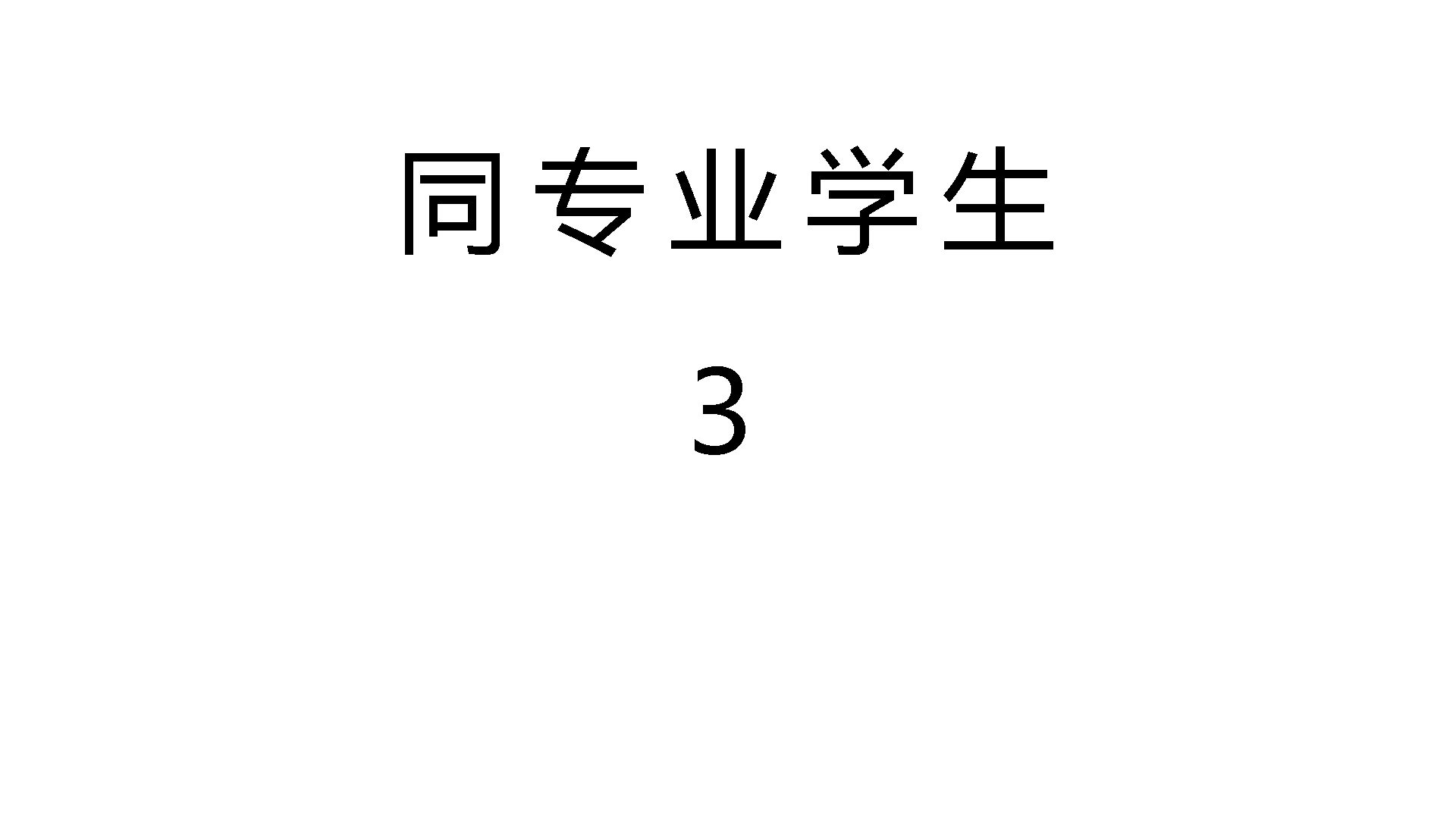

Supplement: Supplementary file 1 [file Data_Sheet_1.ZIP › Stimulus/major3.jpg]

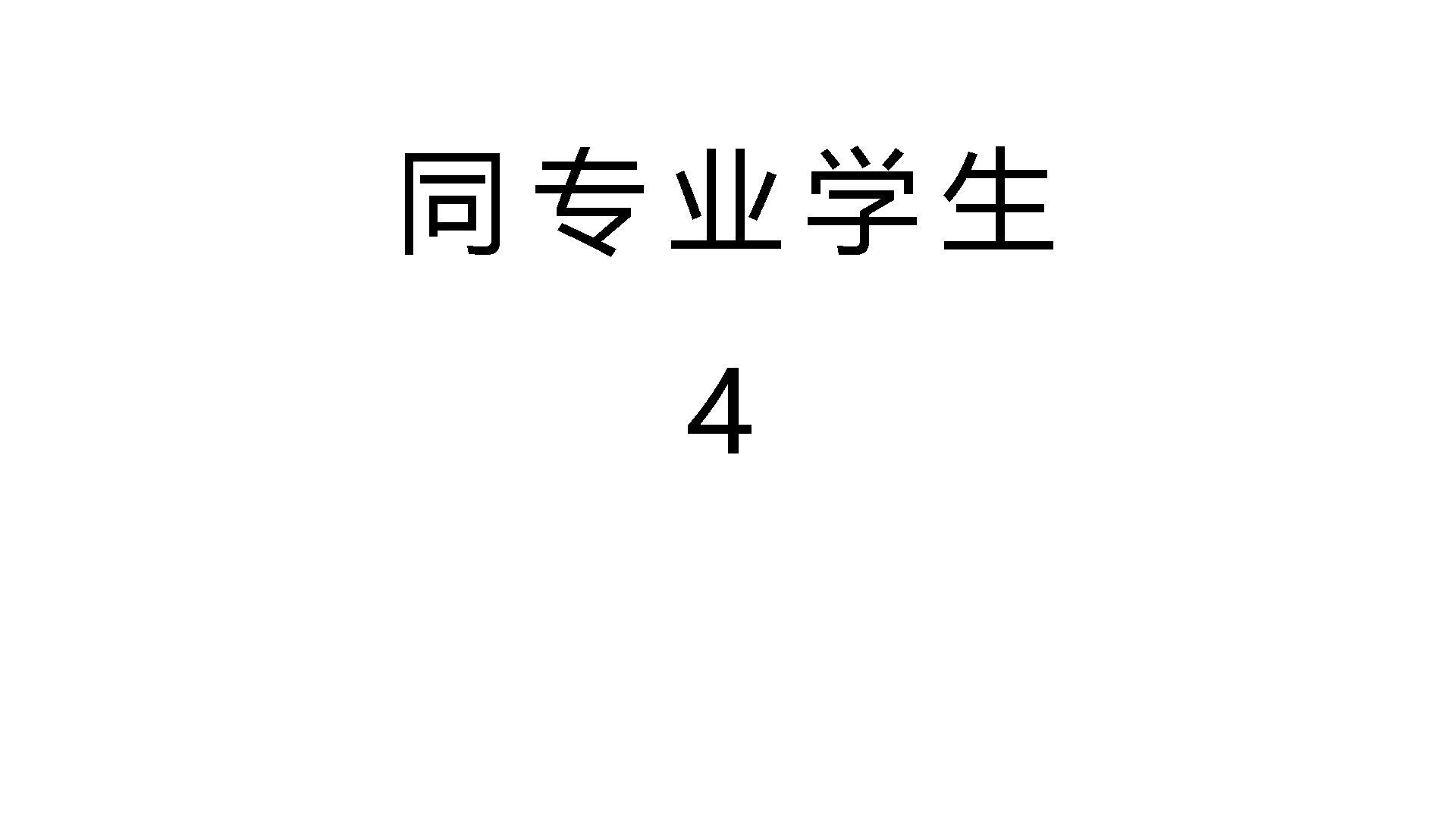

Supplement: Supplementary file 1 [file Data_Sheet_1.ZIP › Stimulus/major4.jpg]

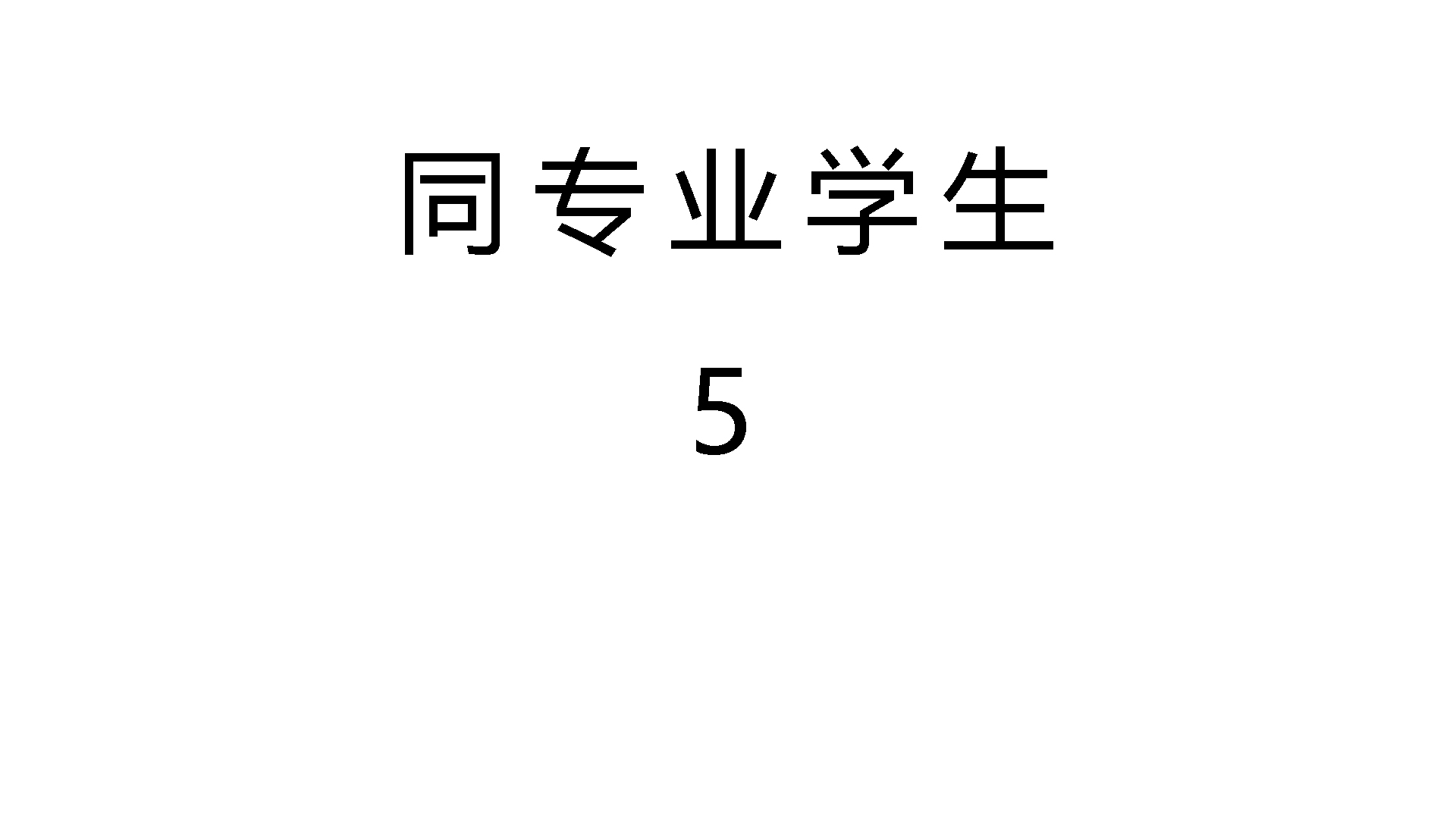

Supplement: Supplementary file 1 [file Data_Sheet_1.ZIP › Stimulus/major5.jpg]

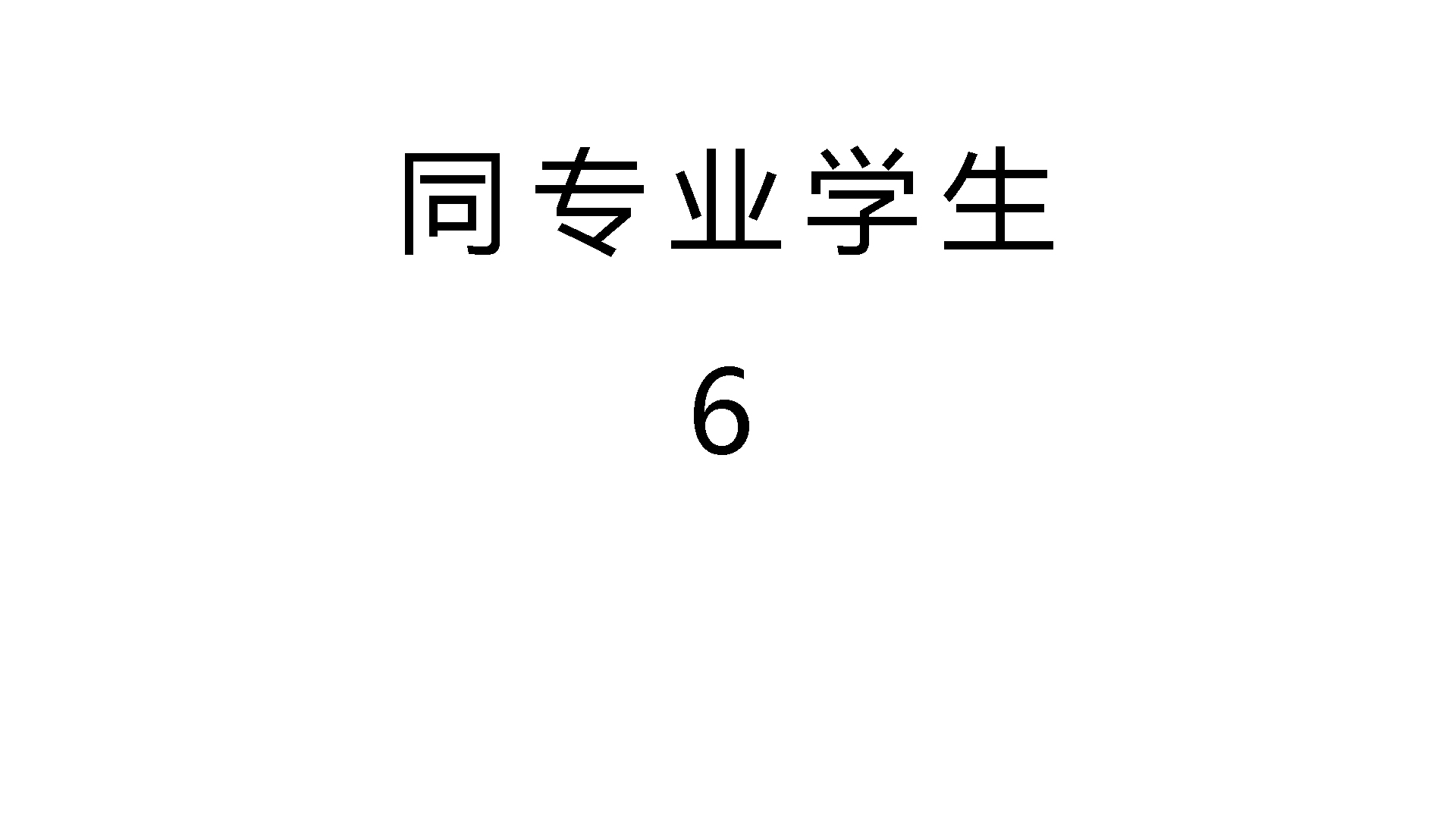

Supplement: Supplementary file 1 [file Data_Sheet_1.ZIP › Stimulus/major6.jpg]

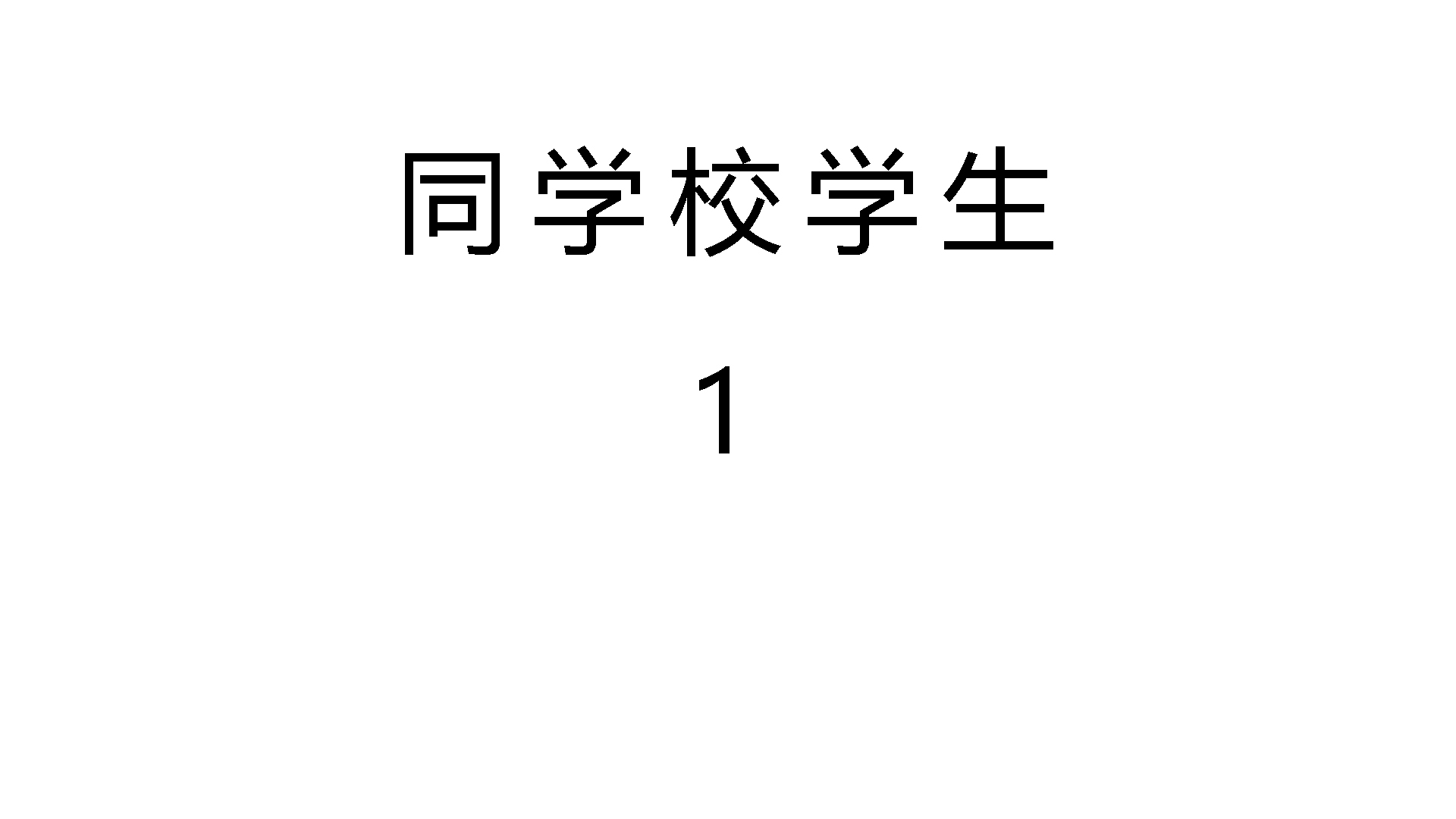

Supplement: Supplementary file 1 [file Data_Sheet_1.ZIP › Stimulus/school1.jpg]

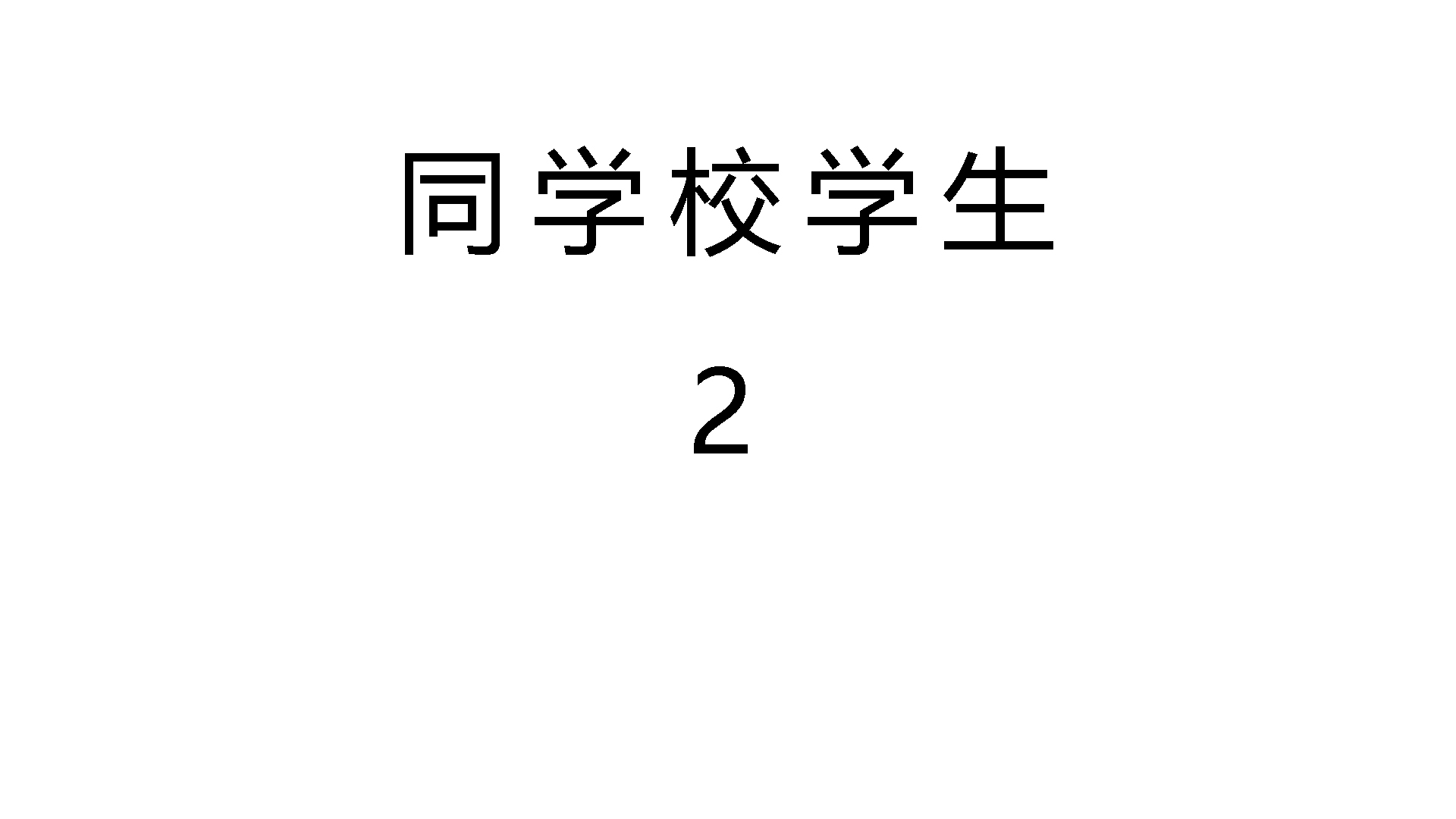

Supplement: Supplementary file 1 [file Data_Sheet_1.ZIP › Stimulus/school2.jpg]

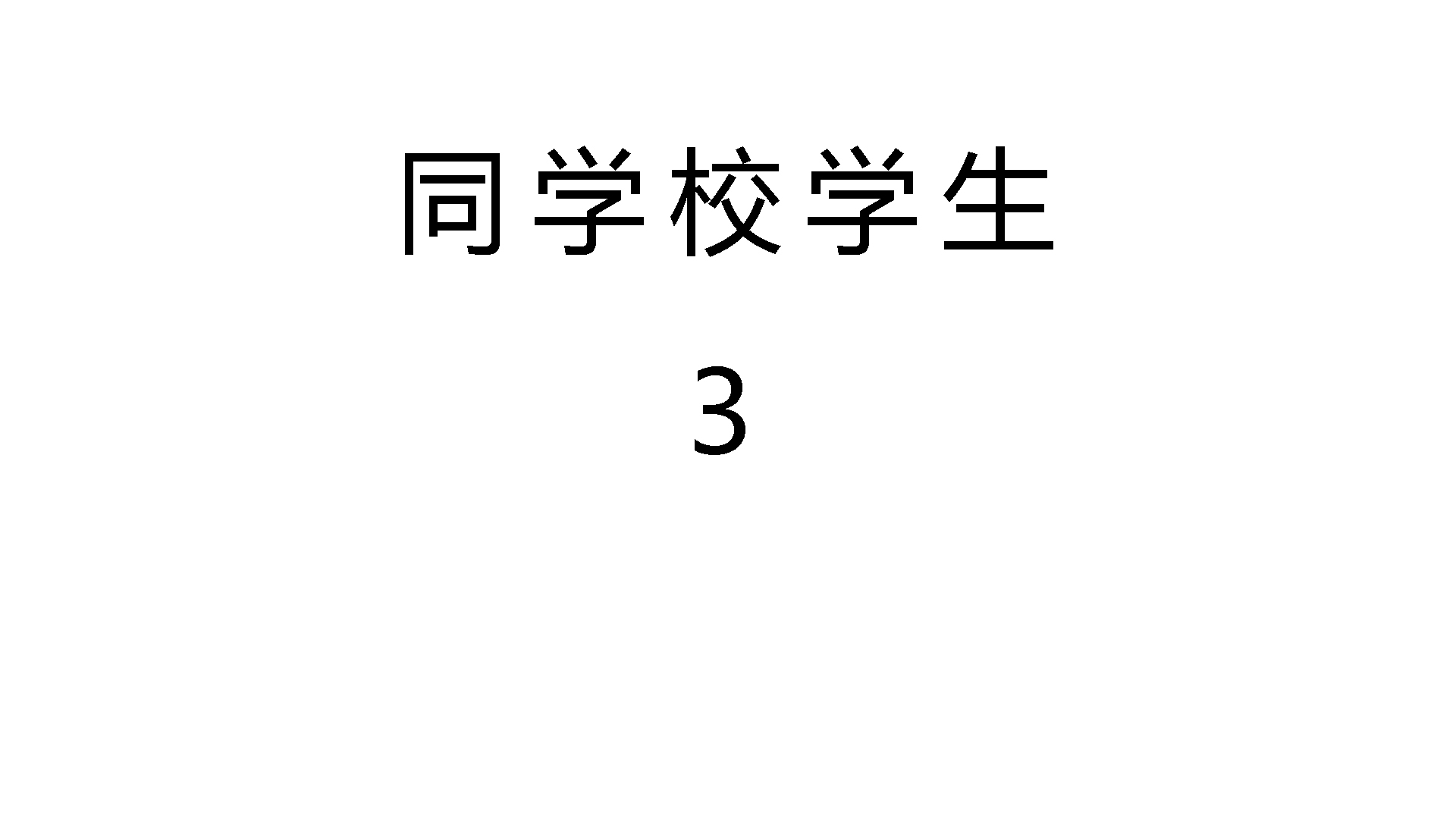

Supplement: Supplementary file 1 [file Data_Sheet_1.ZIP › Stimulus/school3.jpg]

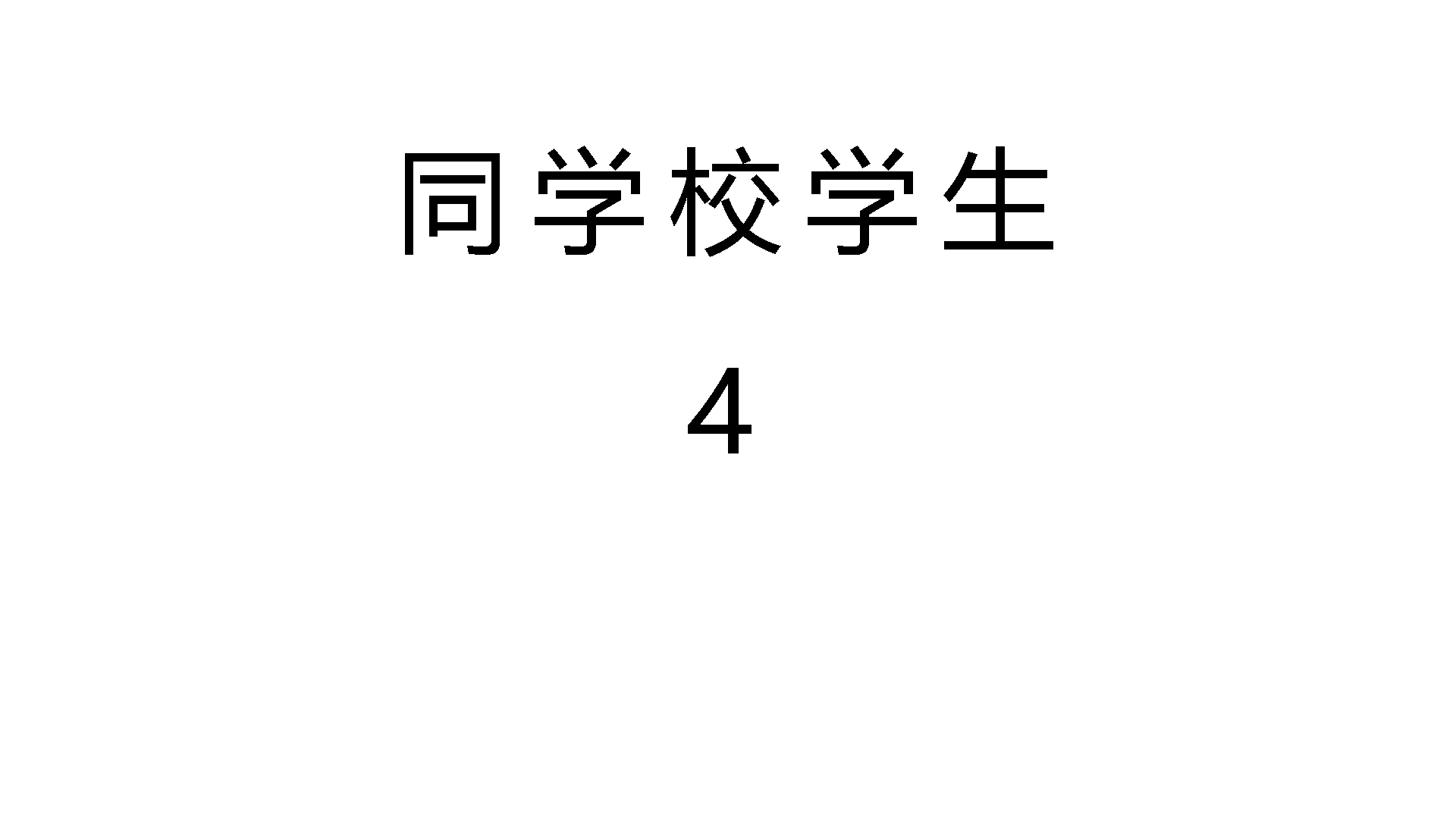

Supplement: Supplementary file 1 [file Data_Sheet_1.ZIP › Stimulus/school4.jpg]

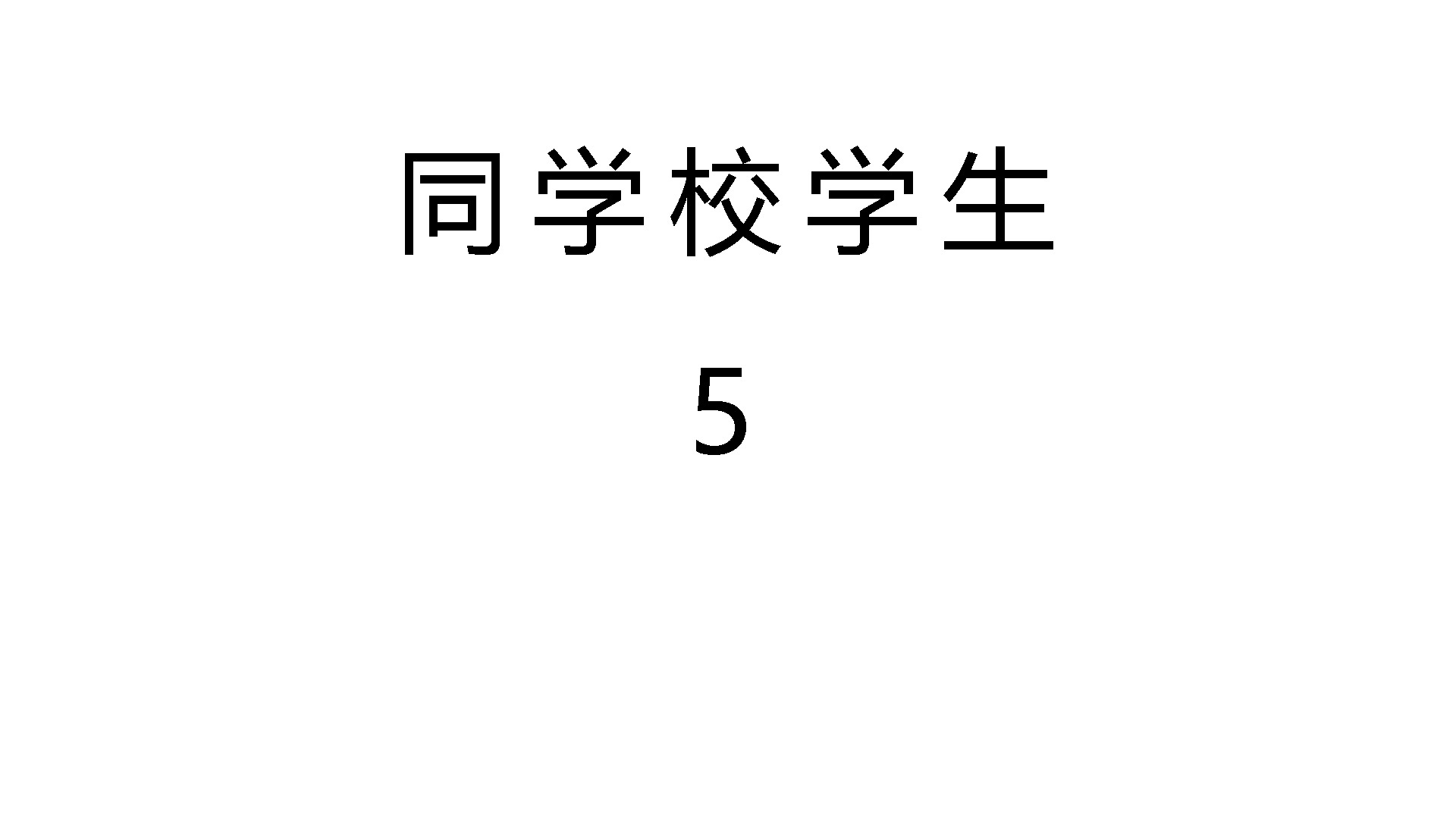

Supplement: Supplementary file 1 [file Data_Sheet_1.ZIP › Stimulus/school5.jpg]

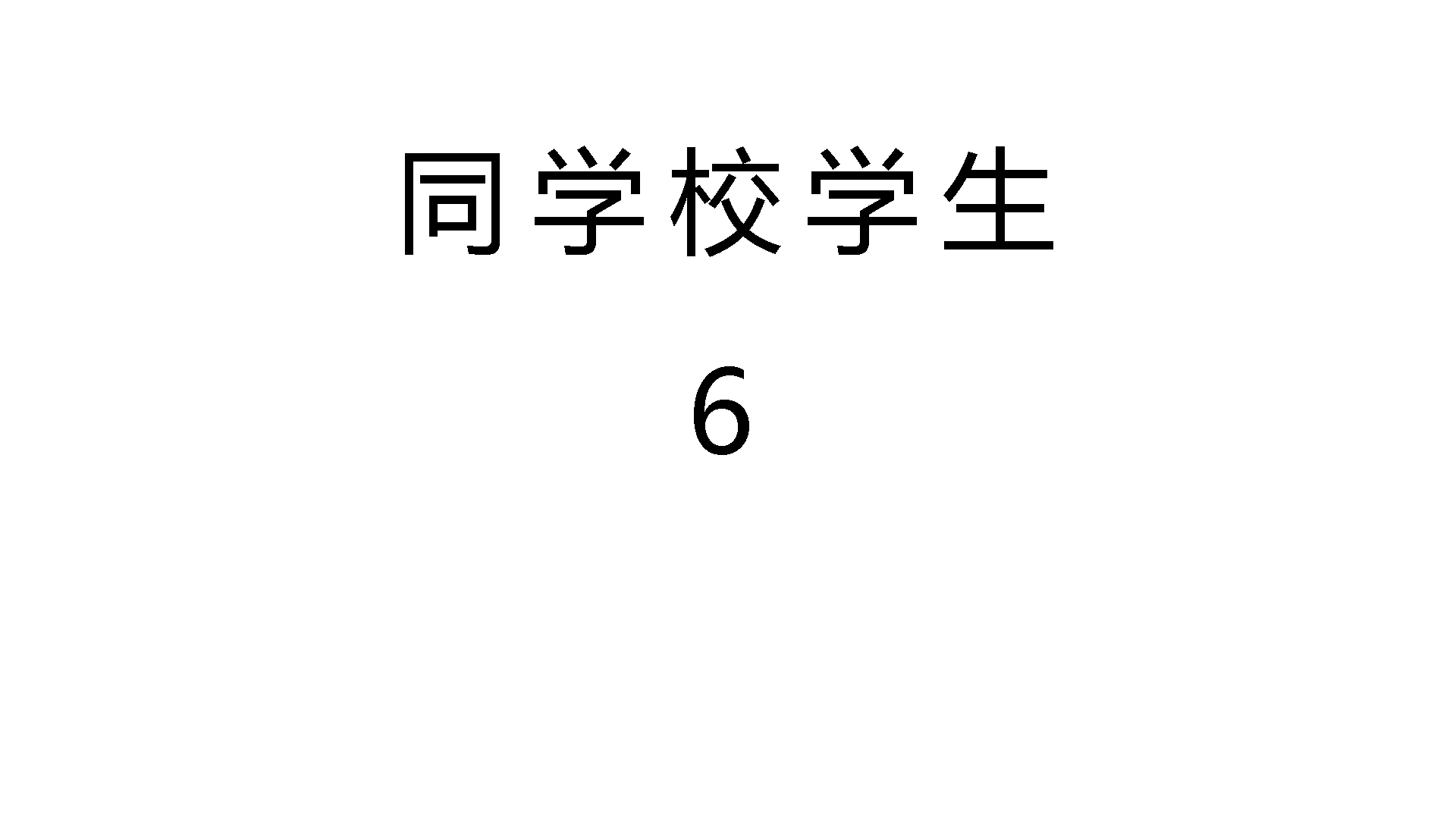

Supplement: Supplementary file 1 [file Data_Sheet_1.ZIP › Stimulus/school6.jpg]

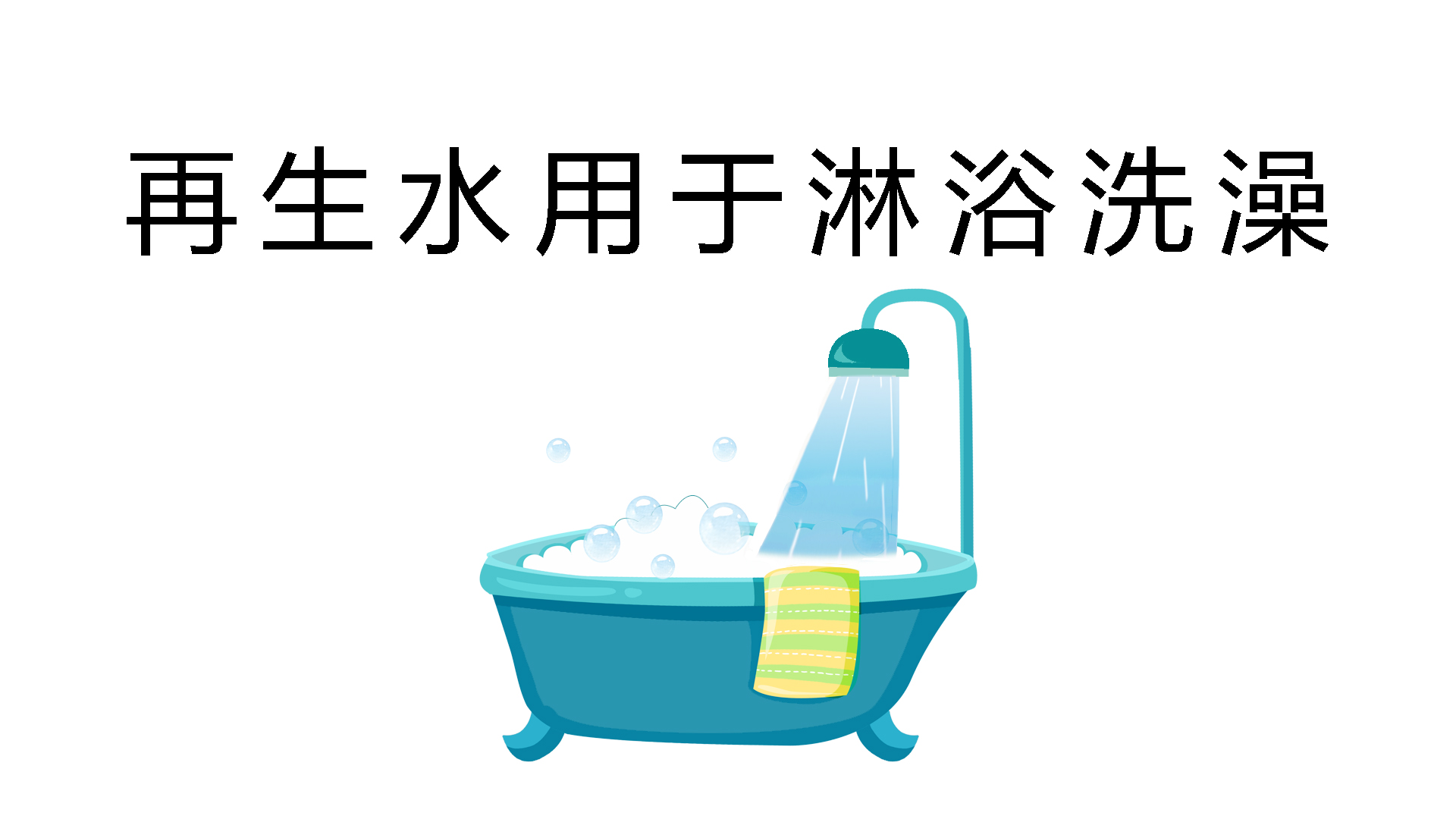

Supplement: Supplementary file 1 [file Data_Sheet_1.ZIP › Stimulus/shower.jpg]

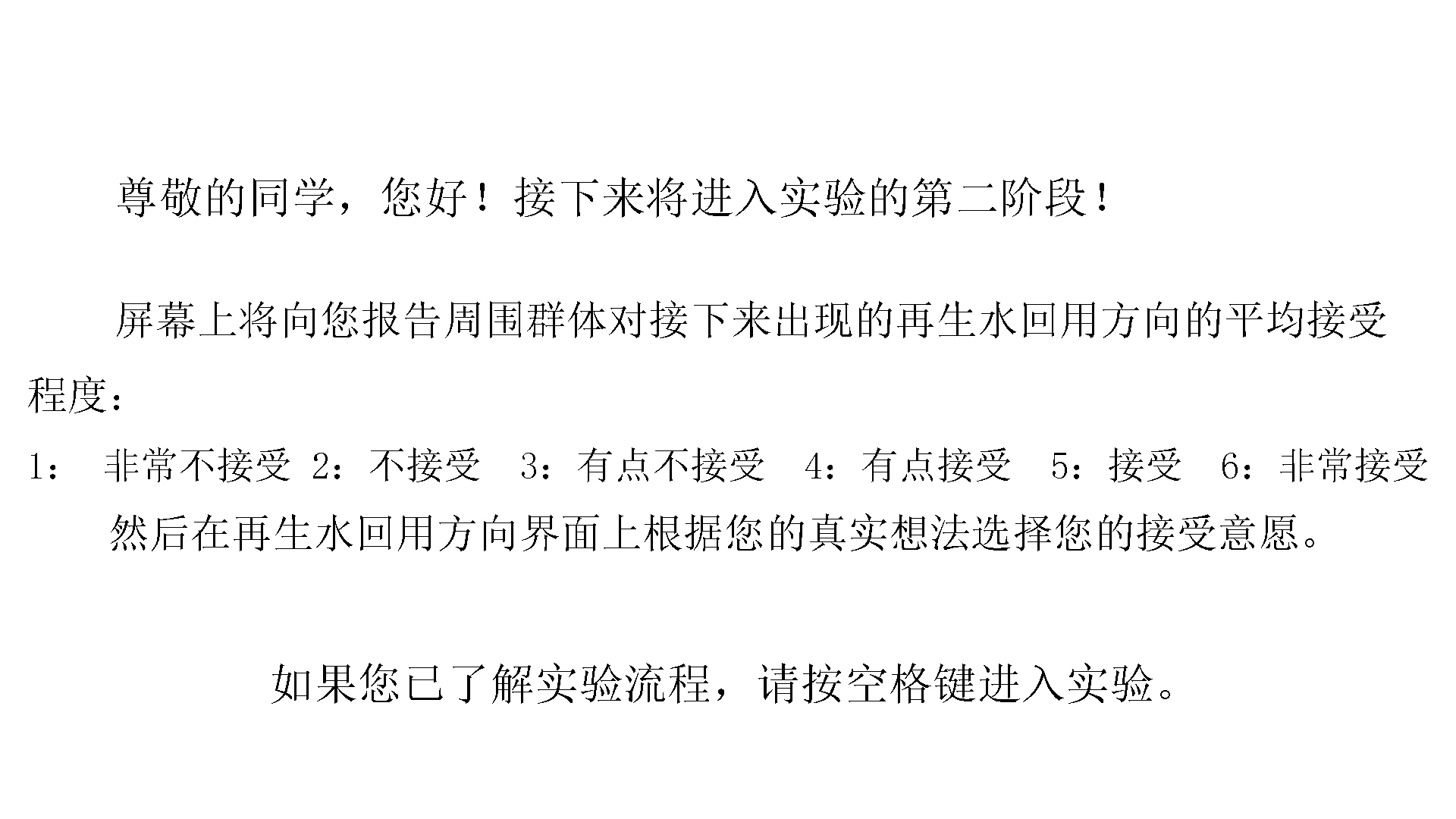

Supplement: Supplementary file 1 [file Data_Sheet_1.ZIP › Stimulus/TASK2.jpg]

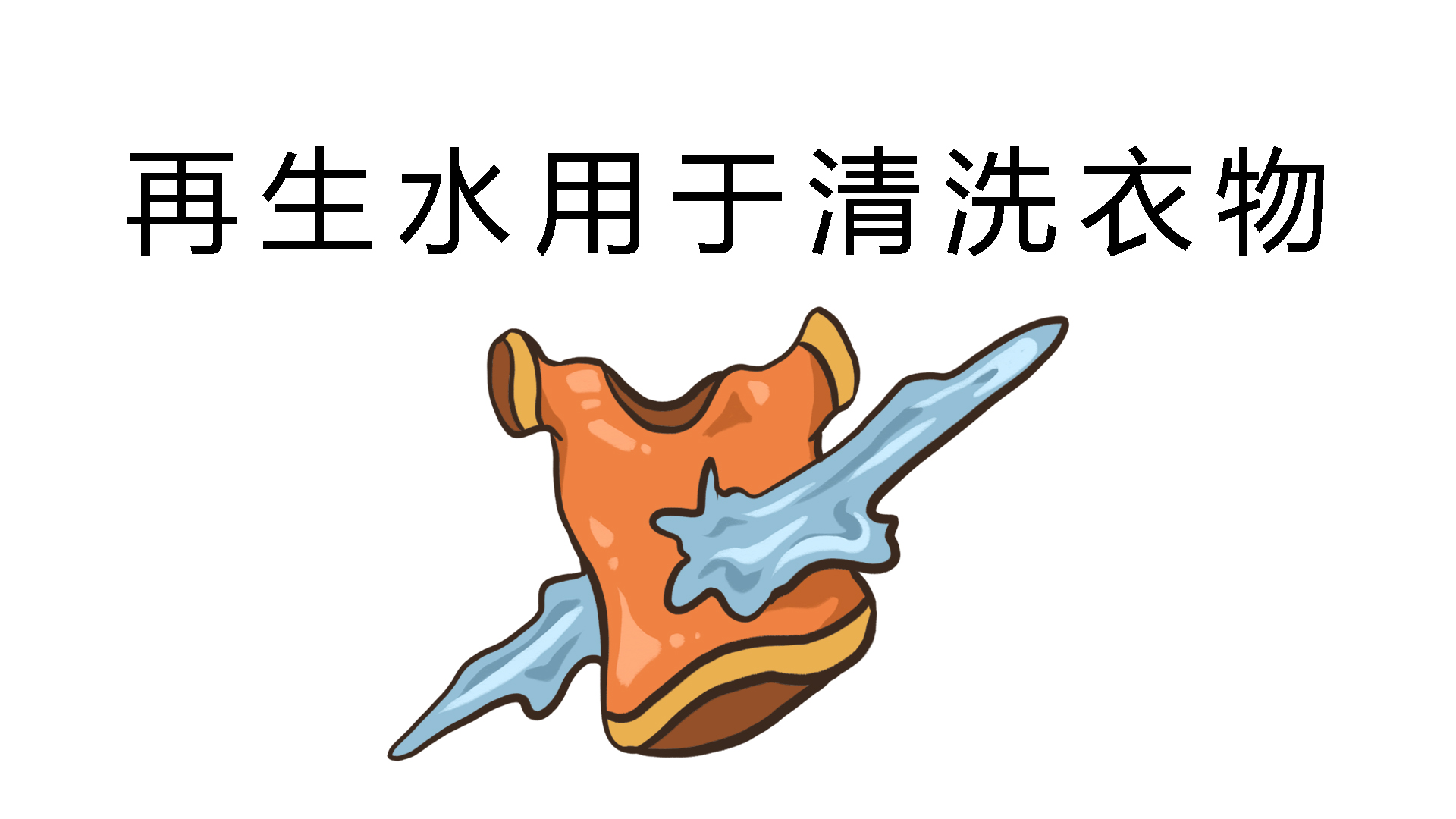

Supplement: Supplementary file 1 [file Data_Sheet_1.ZIP › Stimulus/WashClothes.jpg]

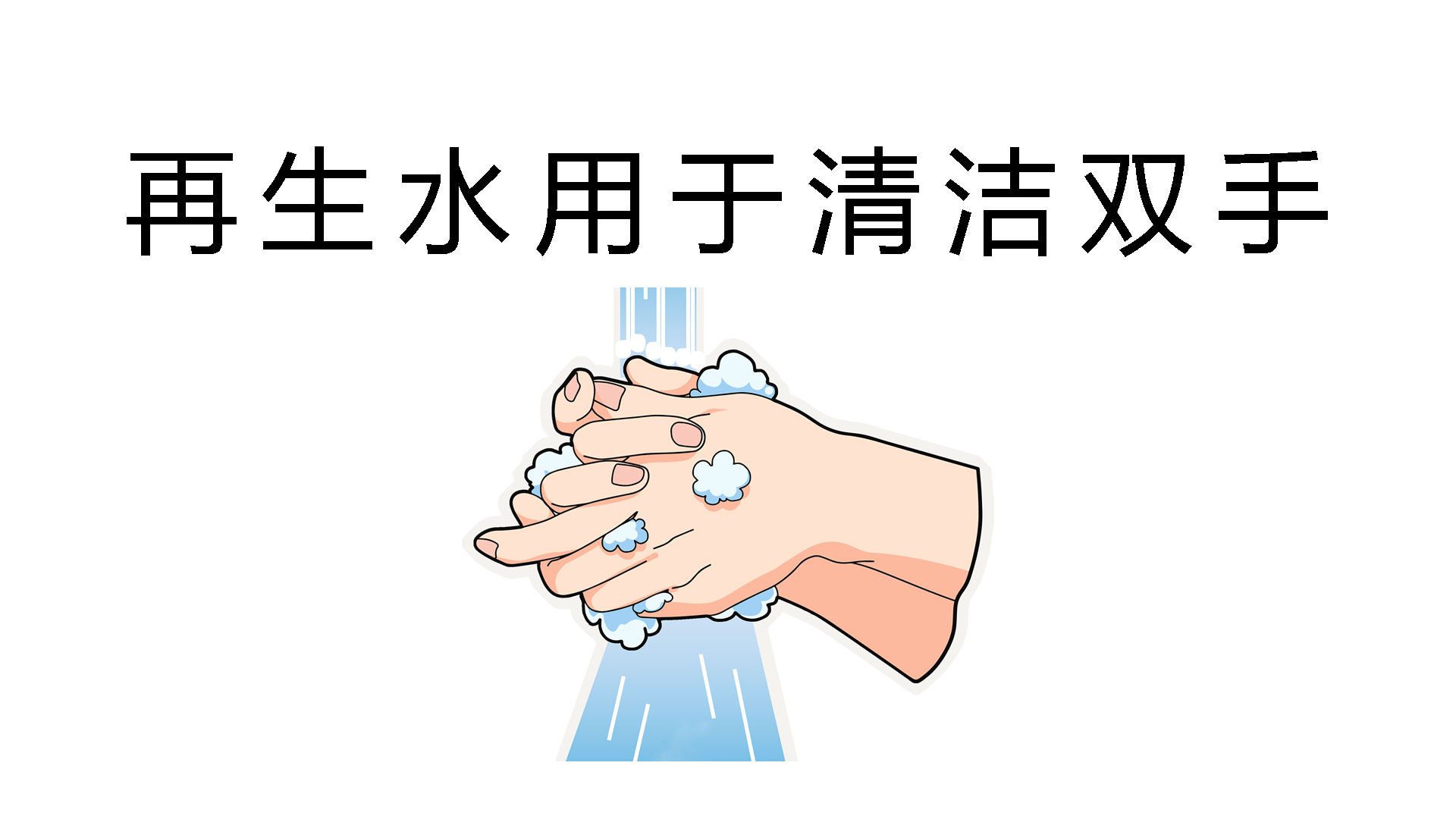

Supplement: Supplementary file 1 [file Data_Sheet_1.ZIP › Stimulus/WashHands.jpg]

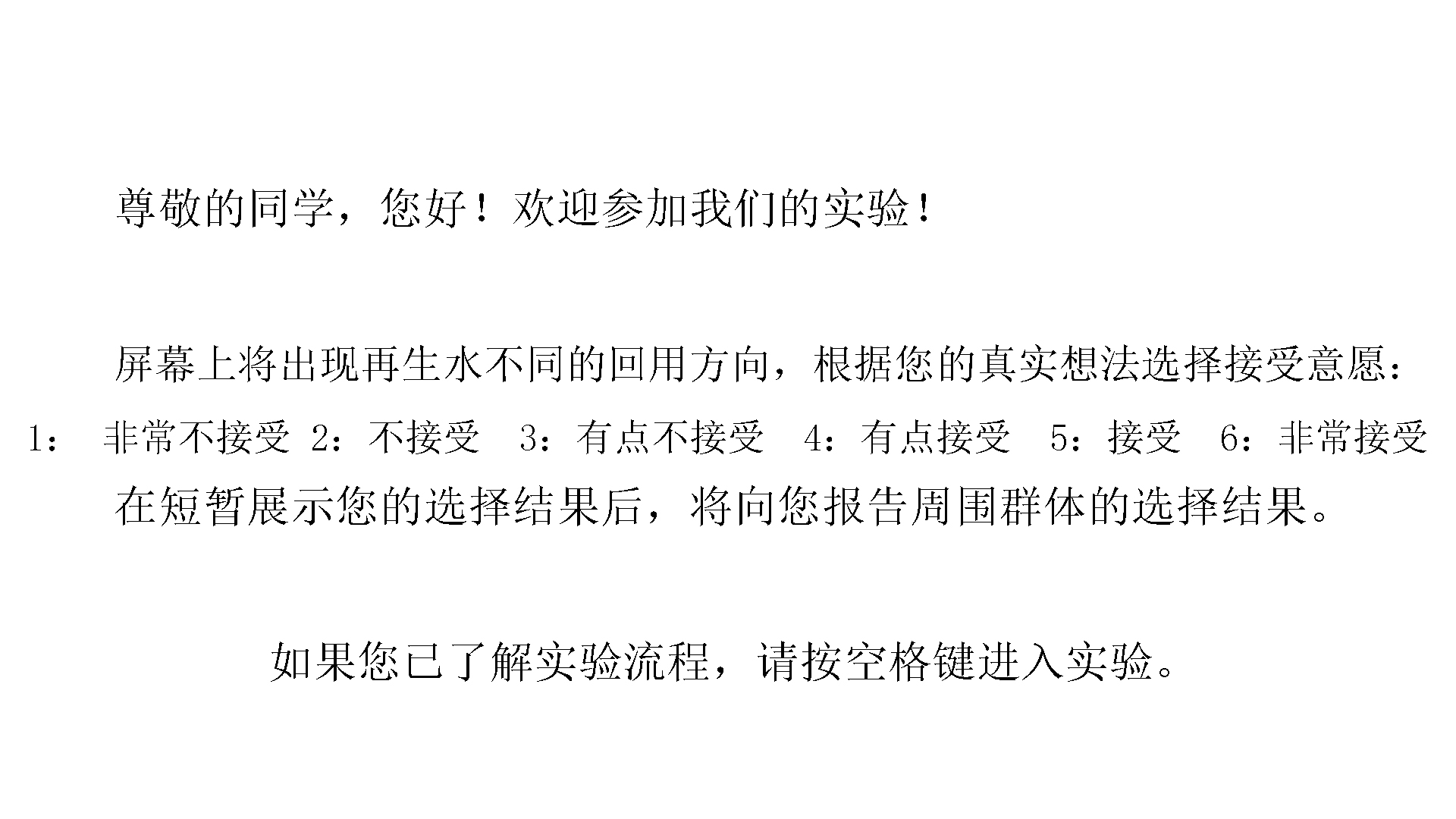

Supplement: Supplementary file 1 [file Data_Sheet_1.ZIP › Stimulus/zhidaoyu.jpg]
